# Casiopeinas of Third Generations: Synthesis, Characterization, Cytotoxic Activity and Structure–Activity Relationships of Mixed Chelate Compounds with Bioactive Secondary Ligands

**DOI:** 10.3390/molecules27113504

**Published:** 2022-05-30

**Authors:** Yeshenia Figueroa-DePaz, Jaime Pérez-Villanueva, Olivia Soria-Arteche, Diego Martínez-Otero, Virginia Gómez-Vidales, Luis Ortiz-Frade, Lena Ruiz-Azuara

**Affiliations:** 1 Departamento de Química Inorgánica y Nuclear, Facultad de Química, Universidad Nacional Autónoma de México, Avenida Universidad 3000, Mexico City 04510, Mexico; yeshita_19@hotmail.com; 2 Departamento de Sistemas Biológicos, División de Ciencias Biológicas y de la Salud, Universidad Autónoma Metropollitana-Xochimilco (UAM-X), Mexico City 04960, Mexico; jpvillanueva@correo.xoc.uam.mx (J.P.-V.); soriao00@gmail.com (O.S.-A.); 3 Centro Conjunto de Investigación en Química Sustentable UAEM-UNAM, Carretera Toluca-Atlacomulco, Km.14.5, Toluca 50200, Mexico; diegomtz@unam.mx; 4 Instituto de Química, Universidad Nacional Autónoma de México, Av. Universidad 3000, C.U., Mexico City 04510, Mexico; gomvidal@gmail.com; 5 Centro de Investigación y Desarrollo Tecnológico en Electroquímica S.C. Parque Tecnológico Querétaro, Sanfandila, Pedro de Escobedo, Querétaro 76703, Mexico; laofrade@gmail.com

**Keywords:** metallodrugs, Casiopeinas, copper, curcumin, dimethoxycurcumin, cytotoxic activity, redox potential, DFT

## Abstract

Casiopeinas are a family of copper(II) coordination compounds that have shown an important antineoplastic effect and low toxicity in normal cells. These compounds induce death cells by apoptosis through a catalytic redox process with endogenous reducing agents. Further studies included a structural variation, improving the activity and selectivity in cancer cells or other targets. In the present work we report the third generation, which contains a bioactive monocharged secondary ligand, as well as the design, synthesis, characterization and antiproliferative activity, of sixteen new copper(II) coordination compounds with curcumin or dimethoxycurcumin as secondary ligands. All compounds were characterized by elemental analysis, FTIR, UV-Vis, magnetic susceptibility, mass spectra with MALDI-flight time, cyclic voltammetry, electron paramagnetic resonance (EPR) spectroscopy and X-ray diffraction. Crystallization of two complexes was achieved in dimethylsulfoxide (DMSO) with polar solvent, and crystal data demonstrated that a square-based or square-base pyramid geometry are possible. A 1:1:1 stoichiometry (diimine: copper: curcuminoid) ratio and the possibility of a nitrate ion as a counterion were supported. ^1^H, ^13^C NMR spectra were used for the ligands. A sulforhodamine B assay was used to evaluate the cytotoxicity effect against two human cancer cell lines, SKLU-1 and HeLa. Electronic descriptors and redox potential were obtained by DFT calculations. Structure–activity relationships are strongly determined by the redox potential (E_1/2_) of copper(II) and molar volume (V) of the complexes. These compounds can be used as a template to open a wide field of research both experimentally and theoretically.

## 1. Introduction

Historically, design of coordination compounds has led to the synthesis, characterization and biological evaluation of new molecules. In this sense, rare earth [1] and transition metals [2,3,4,5], as a structural part of the molecule, are used for different purposes. Prominent examples include the ability to participate in enzymatic and redox processes [6,7] and interactions with various biological targets for specific biological activity. The design of new molecules has addressed various pathologies, such as Alzheimer’s disease [8], Parkinson’s disease [9] and cancer [10], to name a few.

According to the World Health Organization (WHO) [11], in 2020, almost 10 million people worldwide are estimated to have died from cancer, both men and women, making cancer the second leading cause of death from non-communicable disease (NCD). Therefore, research groups have focused on the design of coordination compounds with good biological activity and selectivity against different types of cancer. Cisplatin [12] is a well-known coordination compound that is already commercialized, and its derivatives, such as carboplatin, oxaliplatin, nedaplatin and lobaplatin, are used clinically for the treatment of many types of cancer, such as prostate and colorectal cancer [13]. However, these treatments present severe side effects, including peripheral neuropathies, immunosuppression, nephrotoxicity and ototoxicity, among others [12].

Casiopeinas has emerged as an alternative to improve biological activity with respect to commercial compounds and reduce or eliminate adverse effects. Casiopeinas were synthesized for the first time in 1987 [14,15] as mixed chelate copper(II) compounds presenting an essential metal, given their presence in the body, as well as ligands that modify the redox properties of the metal; these follows a general molecular formula, [Cu(N-N)(O-O)]NO_3_ and [Cu(N-N)(N-O)]NO_3_, where N-N is 2,2′-bipyridines or 1,10-phenanthrolines with or without substituents (primary ligands), O-O is acetylacetonate or salycilaldehydates and N-O can be aminoacidates or peptidates (secondary ligands). Casiopeinas have shown good cytotoxic activity against several cancer cell lines and antineoplastic activity in vitro and in vivo [16,17]. Several mechanisms of action have been proposed to explain these activities, including genotoxicity as a result of DNA interaction [18,19] and overproduction of reactive oxygen species (ROS) [20,21] mediated by endogenous reducers [22] and mitochondrial imbalance [23,24]. On the contrary, it has been shown that Casiopeinas do not present considerable cytotoxic effects in normal cells, which highlights them as potential antitumor agents; in fact, one molecule is already being tested in a clinical phase 1 trial in Mexico.

Due to favorable biological results presented by Casiopeinas against cancer line cells, a quantitative structure–activity relationship (QSAR) study [25] was implemented wherein it was observed that first, the phenanthroline ligand increases the biological activity of these molecules and, second, redox potential is related to their cytotoxic activity and is regulated by the secondary ligand (N-O and O-O). In view of these observations, we designed a third generation of Casiopeinas with a bioactive substance used as a second ligand. Our hypothesis is that the addition of bioactive molecule will increase the antiproliferative activity against various lines of cancer. More interestingly, we chose curcumin as a secondary ligand. Curcumin is a heptanoid compound and the principal bioactive substance presented in a perennial herb known as *Curcuma longa* [26].

The structure of curcumin (1,7-bis(4-hydroxy-3-methoxyphenyl)-1,6-heptadien-3,5-dione) contains an α,β-unsaturated diketonic group. It can be used a polydentate ligand to chelate metals such as copper(II). On the other hand, curcumin exhibits a wide spectrum of biological activities, such as anti-inflammatory, antioxidant [27], antiviral [28], antimicrobial [29] and anticancer activity [30,31,32]. Furthermore, beneficial activity has been observed against various neurodegenerative pathologies, such as Parkinson’s disease and Alzheimer’s disease [33], which makes curcumin a possible treatment. However, oral administration of curcumin has been highlighted as a major problem, and its bioavailability is low due poor gastrointestinal absorption, fast elimination and low aqueous solubility in systemic circulation [34,35]. Curcumin is of particular interest in the pharmaceutical field because of its low toxicity; phase 1 studies show that it is not toxic in humans, even at high doses of 8 g/day, and evidence its chemotherapeutic effects [31].

The low aqueous solubility, as well as the chemical and metabolic instability, of curcumin [36] has led to the synthesis of curcumin analogs in order to increase its solubility [37] and improve its bioavailability. An example of this is dimethoxycurcumin [38], a dimethoxylated analog in -ortho and -para position with respect to the heptanoic chain; studies have shown that its cytotoxic activity against cell lines (such as SKLU-1, HCT-15 and K562, among others) is increased with relative to curcumin [37,39], as well as improvement in terms of water solubility and polar solvents (MeOH, EtOH). Another way to improve the bioavailability of curcumin is to generate coordination compounds with curcumin or analogs (curcuminoids). In fact, there are homoleptic and mixed metal compounds with metals such as Zn [40,41], Mg [39,42], Gd [43,44,45], Fe [46], Ni [47] and Cu [48,49,50,51]; however, characterization of such metal complexes by X-ray is scarce due to their low solubility.

Therefore, the main objective of this work is to synthesize, characterize and evaluate third-generation Casiopeinas in vitro, i.e., ternary copper(II) compounds, with bipyridines and phenanthrolines substituted with different numbers of methyl groups as first ligands and a bioactive substance (curcumin or dimethoxycurcumin) as secondary ligands to increase curcumin’s solubility and improve biological activity and selectivity against cancer cell lines compared to drugs for clinical use. Additionally, several DFT studies were carried out to obtain electronic descriptors, such as molar volume (V), electron affinity (EA), ionization energy (IE) and half-wave potential (E_1/2_) to search for structure–activity relationships. The results presented here can generate physicochemical studies, in vitro and in vivo evaluations or studies to determine the mechanism of action of this type of compound.

## 2. Results and Discussion

### 2.1. Synthesis of Curcumin (L1) and Dimethoxycurcumin (L2) Ligands

**Figure 1:**
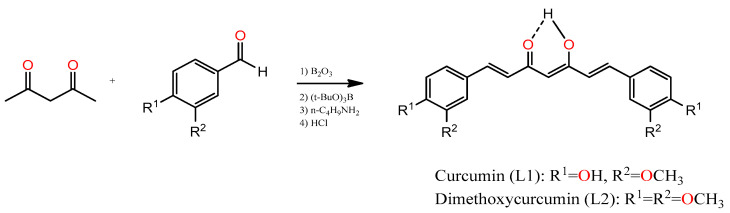
Synthesis of curcumin (L1) and dimethoxycurcumin (L2).

L1 and L2 were synthesized by condensation of a benzaldehyde molecule with acetylacetone (acac) under an N_2_ atmosphere. Heptanoids were obtained in moderate yields. The structures of heptanoids were confirmed by melting points, elemental analysis, FT-IR and mass spectra. ^1^H, ^13^C NMR data of curcumin and dimethoxycurcumin indicate both molecules exist almost in the enol form in solution, for which the spectrum in DMSO-d^6^ (Figures S1–S7) displays singlets at 16.40 and 16.29 ppm. Theses protons are involved in an intramolecular hydrogen bridge, and the methine proton exhibits one singlet at 6.06 and 6.11 ppm, respectively, in accordance with the structure shown in Figure 1. Curcumin is less soluble in water, methanol and ethanol than dimethoxycurcumin, but both compounds are fairly soluble in DMSO and DFM. UV-vis spectrophotometric analysis of L1 and L2 solutions showed a maximum at 428 and 436 nm, respectively (63,288 and 31,485 L/mol·cm).

### 2.2. Synthesis and Characterization of Copper Complexes [Cu(Dn)(L1)]NO_3_ and [Cu(Dn)(L2)]NO_3_

**Figure 2:**
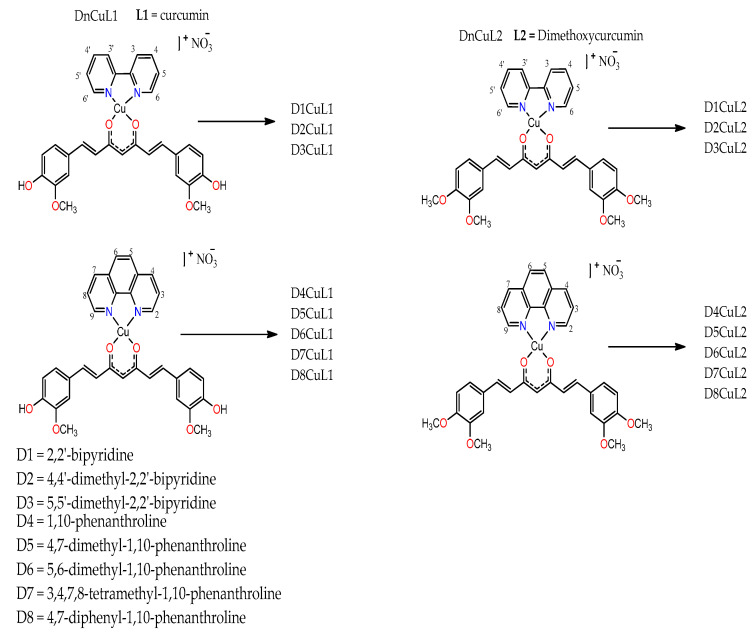
Structure of the studied mixed chelate copper(II) compound.

Sixteen novel ternary heteroleptic complexes of copper(II) of diimine with curcumin or dimethoxycurcumin were synthesized and characterized by elemental analysis, FTIR, conductivity, susceptibility, UV-vis, MALDI-MS and cyclic voltamperometry. Substituents of bipyridine or phenanthroline codes are show in Figure 2. All coordination compounds of copper(II) are slightly more soluble than free ligands (curcumin and dimethoxycurcumin) in water, methanol and ethanol and fairly soluble in DMSO and DMF. Therefore, we chose dimethylsulfoxide as solvent to ensure complete solubility of each compound and performed homogeneous characterization.

#### 2.2.1. Molar Conductivity, Magnetic Moments and Electron Paramagnetic Resonance (EPR) Spectroscopy

According to Greenwood [52], molar conductivity values in the range of 50–70 µS are common in type 1:1 electrolytes in DMSO at 25 °C; these values are widely used in the literature. The only compound that showed a value in this range is D6CuL1 (59.1 µS), which indicates that it is a 1:1 electrolyte. However, most compounds presented values within the range of 23.2–37.4 µS; these values are below those reported by Greenwood. Low molar conductivity does necessarily mean that complexes are not ionic species; in fact, all molecules conduct electricity but only moderately, suggesting that the ionic nature of type 1:1 electrolytes is weak, and in solution, the nitrate ion acts as a counterion. We propose that these results indicate that a small fraction of the compound presents dissociation. On the other hand, values lower than 50 µS could be explained by the low ionic mobility of the complex cation entity with +1 charge, which could be the cause of the low observed electrolytic conductivity. Geary [53] reported data showing coordination complexes with low electrical conductivity values (35 µS). These results are interesting because although there are reports of the synthesis of mixed copper(II) coordination compounds that contain curcumin or analogs within their structure, it is not common for authors to use this technique for their characterization. There are few reports of the molar conductivity values of mixed copper(II) compound in water [54] or DMF [55,56,57]. On the other hand, there are conductivity reports for some copper(II) coordination compounds wherein non-ionic complexes were obtained and electrical conductivity data in DMSO were reported, with values between 6 and 11 μS, which indicates that these are non-electrolyte species [58]. Therefore, our compounds show a small dissociation of an NO_3_ counterion.

Magnetic susceptibility values of the complexes allow for determination of the effective magnetic moment (μeff) [59]. Most of the coordination compounds exhibited values between 1.79 and 2.3 BM, corresponding to the one unpaired electron characteristic of paramagnetic molecules that contain copper(II) of configuration d^9^, whereas five coordination compounds with curcumin as secondary ligand showed low values of μ_eff_ due to antiferromagnetic coupling between two unpaired electrons in each copper atom of two different molecules.

**Figure 3:**
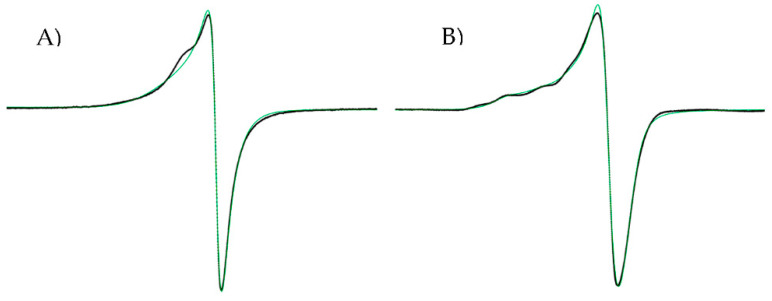
EPR spectra. (**A**) Complexed D7CuL1 and (**B**) complexed D6CuL2.

In general, the solid-state EPR spectra of mixed chelated copper(II) coordination compounds exhibited axial symmetry, which is typical of a d^9^ configuration in a fundamental state with hyperfine and superhyperfine couplings (Figure 3). The g values calculated for the studied copper(II) complexes are within the range of 2.04–2.09 (Table S1), as g_II_ > g_I_, which is consistent with an unpaired electron in the dx^2^–dy^2^ orbital, i.e., coordination geometries corresponding to a square planar or square-based pyramid [60,61]. Values of g_II_ <2.3 suggest a covalent environment [62] around for the copper in the complexes and that heteroatoms of ligands (diimine and heptanoids) interact strongly with copper atoms.

#### 2.2.2. Infrared Spectra

The characteristic bands of curcumin include the O-H stretching vibration of the phenol group observed at 3438 cm^−1^, a band at 1628 cm^−1^ due to the intramolecular hydrogen bond of the enol and a band at 1510 cm^−1^ assigned to the C=C stretching vibration of the vinyl bond. Dimethoxycurcumin shows a similar spectrum to that of curcumin; the difference is that the stretching vibration band of methyl and methylene groups at 2946 cm^−1^ is more evident because a proton is exchanged for a methyl group. On the other hand, the modifications observed in the spectra of all mixed coordination compounds are very similar. The most considerable difference is in the displacements observed in the vibration band (C=O) with respect to secondary ligands (1627 cm^−1^) described below. A band was found between 1576 and 1599 cm^−1^ corresponding to the C-O vibration in the enol, which indicates that coordination of the ligands with the metal occurred. A new band at 1384 cm^−1^ was found in the spectrum of coordination compounds corresponding to the nitrate group as a counterion. Other bands appeared around 478 cm^−1^ due to M-O vibration, suggesting that the interaction between copper and oxygen atoms of β-diketone is involved in the coordination sphere.

#### 2.2.3. UV-Vis Spectrophotometric Analysis

**Figure 4:**
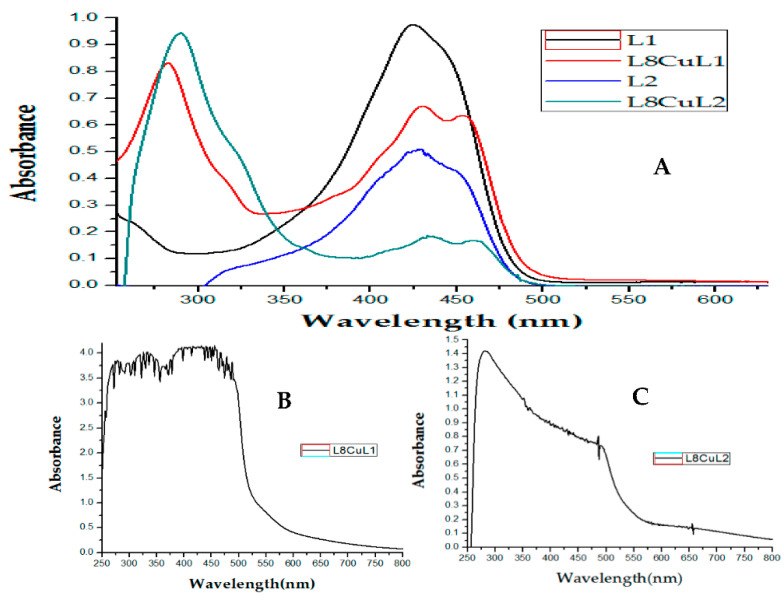
(**A**) Electronic absorption spectra of L1 (15 μM), L2 (15 μM), D8CuL1 (15 μM) and D8CuL2 (15 μM); (**B**) d-d transitions for D8CuL1 (2 mM); (**C**) d-d transitions for D8CuL2 (2 mM).

UV-vis absorption spectra of ligands L1 and L2 showed a main band at 424 nm and 427 nm, respectively, due to n-π* transitions corresponding to charge transfer effects (Figure 4A). Bands at 260 and 266 nm are assigned to π-π* transitions of the aromatic ring; however, these bands are not evident at the low concentrations used to maintain absorbance values below 1 to employ the Beer-Lambert law and calculate the molar extinction coefficients. In coordination compounds, the electronic spectrum always exhibits threes bands (Table 1); for this reason, we will only discuss one compound from each family of complexes (Figure 4A). The first band occurs at 266–321 nm because π-π* transitions are mediated by the contribution of the aromatic rings of curcuminoids, as well as the addition of aromatic rings of diimine. The second absorption maximum (λ_max_) in the range of 428–436 nm was attributed to the ligand–metal charge transfer due to the bathochromic shift with respect to λ_max_ of curcumin, indicating coordination of the carbonyl group of secondary ligands to copper atoms. It is worth mentioning that these transitions are not those of maximum absorption for D8CuL1 and D8CuL2 compounds, as these transitions have phenyl groups that increased the absorbance of the π-π* transitions (Figure 4A). The third band is observed at values between 448 and 460 nm and can be assigned to metal charge transfer, evidencing an electronic π-backbonding (Figure 4A). The extinction coefficients for the copper(II) complexes are high values, as they are totally allowed by Laporte and spin, giving rise to more intense absorptions, unlike the d-d transitions, which are prohibited by Laporte and generate broad bands as a result of the combination of the three allowed transitions for a square-based pyramid geometry in the region of 555–665 nm (Figure 4B,C), where a water molecule can be coordinated in the axial positions of copper. However, square-based geometry (600–700 nm) is also possible, as water molecules may not coordinate to the copper atom, as observed in the obtained monocrystals.

#### 2.2.4. Mass Spectra

Mass spectroscopic analysis of L1 and L2 ligands ([Ligand + H]^+^) showed a molecular ion peak at *m*/*z* of 369 and 397, respectively, for the last the molecular ion, as well as the base peak, suggesting that the obtained molecule is found in greater abundance; therefore, our purification method is efficient, despite the low synthesis yields. The Table 2 shows the theoretical and obtained molecular ion peaks of copper(II) coordination compounds; the theoretical copper isotopic patterns are in agreement with the experimental results.

For many molecules, it was observed that the molecular ion corresponds to the base peak; these data suggest that the most predominant species is the complex cation of copper(II) ([DnCuLn]^+^) (Figures S8–S15), which is formed by loss of the counterion (NO_3_). On the other hand, the results confirm that the stoichiometry of the copper(II) coordination compounds is 1:1:1 (diimine: Cu: secondary ligand), as determined by elemental analysis.

**Figure 5:**
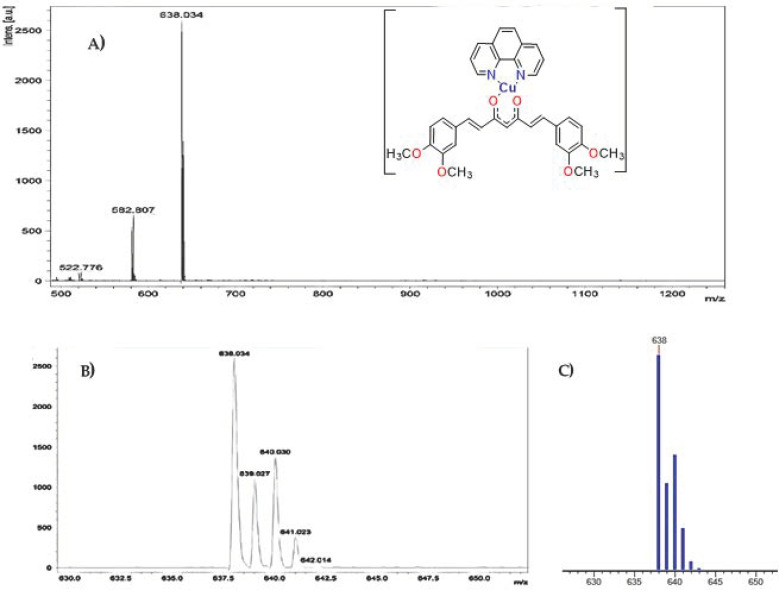
(**A**) MALDI-TOF mass spectra of [L4CuL2]+; (**B**) calculated isotopic spectral pattern of complex theoretical and (**C**) isotopic spectral patterns.

In addition, all coordination compounds with curcumin as secondary ligand exhibited *m*/*z* data, which represent the complex fragments without one or two –OH or –OMe groups over the aromatic rings of curcumin. The same behavior was observed for all complexes with dimethoxycurcumin with the loss of 2 or 4 –OMe groups. Figure 5 shows the behaviors described above for D4CuL2; the peak at *m*/*z* of 638 is assigned to molecular ion [D4CuL2]^+^, and the peaks at *m*/*z* 582 and 522 are assigned to the fragment of the molecule that loses 2 or 4 –OMe groups, respectively.

#### 2.2.5. Cyclic Voltammetry Measurements

**Figure 6:**
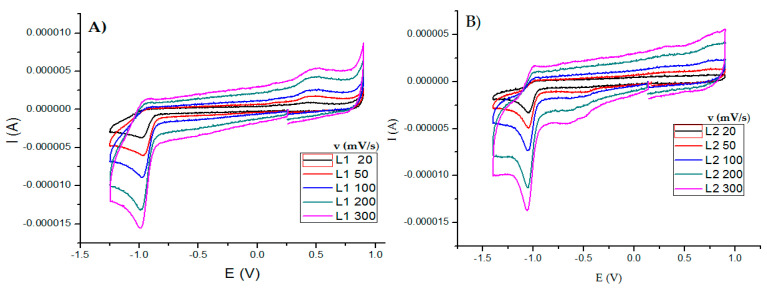
Cyclic voltammograms for 0.001 M ligands in DMSO with 0.1 M tetrabutylammonium hexafluorophosphate. (**A**) Curcumin and (**B**) dimethoxycurcumin. Scan rates in the range of 20–300 mV/s. All experiments were referenced to the pair Fc^+^/Fc.

In this work, the electrochemical behavior of curcuminoids (L1 and L2) was compared with that of copper(II) complexes using DMSO as solvent. All cyclic voltammograms were obtained from open-circuit potential in the negative direction using a potential range of −1.5–1.5 V. Ligands showed a reduction signal Ic at −0.978 V for curcumin (Figure 6A) and −1.05 V for dimethoxycurcumin (Figure 6B). When the scan rate was increased, an oxidation process Ic was observed (Figure 6A), which is typical for an ECi mechanism associated with the reduction C=O moieties, similar to quinone molecules (R-COCHCO + 2H^+^ + 2e^−^ → R-COHCHCOH-R) [63]. Furthermore, curcumin showed a broad oxidation peak Ia at 0.488 V, which is generally assigned a redox process with a slow electrode kinetic associated with phenol oxidation [64,65]. L2 did not show this oxidation process due to the change from –OH to –OCH_3_, which is the main structural difference between these ligands. An inversion study was carried out to correctly associate each redox process for curcuminoids (Figures S16 and S17).

**Figure 7:**
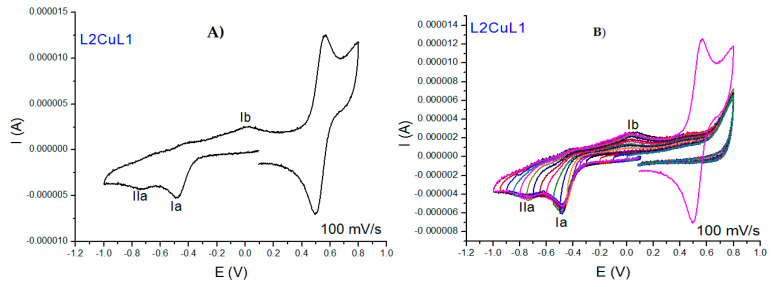
Cyclic voltammograms for 0.001 M of L2CuL1 in DMSO with 0.1 M tetrabutylammonium hexafluorophosphate. (**A**) voltammograms at 100 mV/s and (**B**) inversion study at 100 mV/s. All the experiments were referenced to the pair Fc^+^/Fc.

All cyclic voltammograms of the copper(II) coordination compounds with L1 and L2 are similar to one another; therefore, we will discuss the results of L2CuL1 and L4CuL2 compounds as an example. In general, all complexes exhibited two similar behaviors first; results show that the reduction of ligands does not take place in the complexes due to the coordination with copper; this fact is important because it suggests that generating copper(II) coordination compounds avoids the autoxidation and degradation [36,65] of free ligands, which has been observed in in vivo pharmacokinetic studies [34]. Second, two signals resulting from the metal reduction process were observed. Figure 7A shows that the L2CuL1voltammogram of the first process (Ia) at −0.484 V is caused by the copper(II) reduction in the compound (CuII + 1e^−^ → CuI), and the second process (IIa) at −0.739 V is assigned to the reduction of the copper(I) species generated in the first process (Ia) towards the formation of copper(0). Signal Ib at 0.023 V is associated with copper(I) oxidation (CuI → CuII + 1e^−^). Inversion studies allowed signal Ia to be associated with Ib. Epa, Epc and ΔE were determined at a speed of 100 mV/s (Figure 7B).

**Figure 8:**
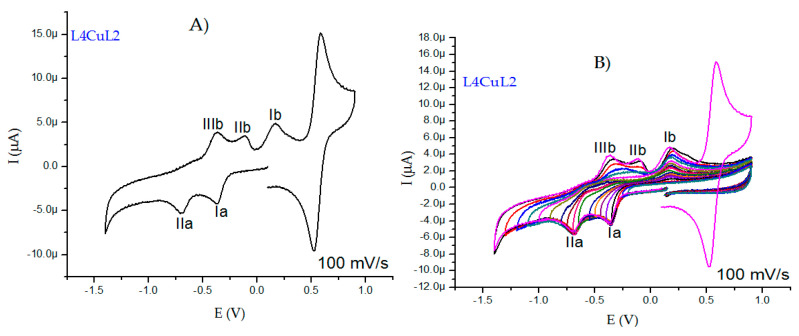
Cyclic voltammograms for 0.001 M of L4CuL2 in DMSO with 0.1 M tetrabutylammonium hexafluorophosphate. (**A**) Voltammograms at 100 mV/s and (**B**) inversion study at 100 mV/s. All the experiments were referenced to the pair Fc^+^/Fc.

Although we have mentioned that the electrochemical behavior is similar for all the compounds studied in this work, it is important that both cathodic peaks are more evident in the family of coordination compounds with L2 (Figure 8A), and the peaks shift to different potentials due to substituents on the aromatic rings of the diimines. Figure 8A shows the L4CuL2 voltammogram, where a cathodic peak at −0.373 V is ascribed to the reduction of the copper atom (CuII + 1e^−^ → CuI), and a second cathodic peak at −0.708 V is associated with the reduction of the copper(I) species to form copper(0) (CuI + 1e^−^ → Cu0). Three anodic peaks (IIIb, IIb and Ib) appearing at −0.364 V, −0.111 V and 0.168 V (Figure 8B), respectively, can be associated to the redissolution of copper(0) species or to the adsorption of ligands being released in the formation of a Cu(0) deposit. Anodic and cathodic processes were associated by inversion studies (Figure 8B).

The process related to the biological activity of the coordination compound of copper(II) is the reduction of Cu(II) to Cu(I) and the reoxidation of Cu(I) to Cu(II) [65]; therefore, the determination of parameters such as Epa, Epc, ΔE and E_1/2_ were terminated only for those processes (Table 3). The separation between Epc and Epa is greater than 60 mV; hence, it was determined that the complexes present a quasi-reversible behavior, i.e., the electronic transfer is slow and is not controlled by the diffusion. This behavior can be attributed to the change in geometry around the metallic center; an example is when a Cu(II) compound with square-based pyramid geometry changes to a Cu(I) compound with a tetrahedral geometry.

In general, coordination compounds with L1 show more negative half-wave potentials and are therefore are easily oxidized compared to coordination compounds with L2 (Table 3). This suggests the possibility that curcumin presents an autoxidation process [65], allowing copper to act as the reducing agent. In coordination compounds with bipyridine, there is an increase in the electron density on the copper atom, so it is difficult for it to gain one electron (more negative values). Complexes with phenanthroline facilitate charge distribution on the aromatic rings and not close to the metal center, so copper could gain one electron (less negative values).

#### 2.2.6. X-ray

**Figure 9:**
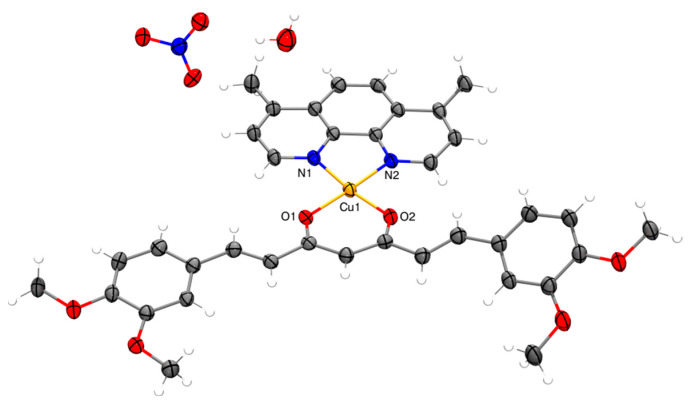
Molecular structure of complex D5CuL2 with displacement ellipsoids at 50% probability level.

**Figure 10:**
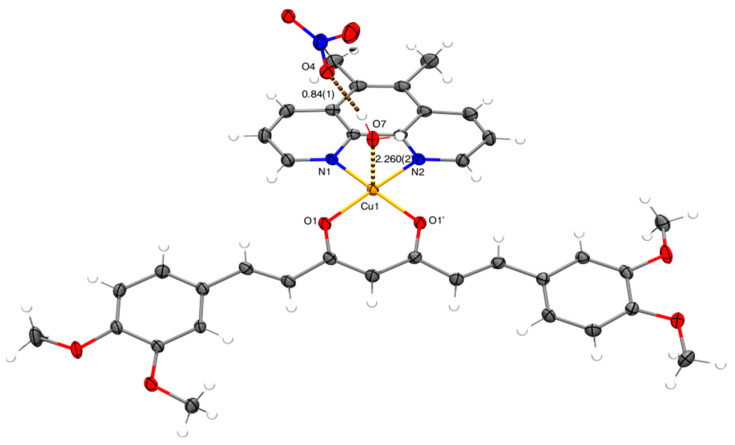
Molecular structure of complex D6CuL2 with displacement ellipsoids at 50% probability level.

Obtaining single crystals of mixed chelate copper(II) coordination compounds with curcuminoids as ligands is complicated due to the low solubility of the complexes, with few reports in the literature [39,66,67]. Small crystals of the analogous complexes D5CuL2 and D6CuL2 were obtained (Figure 9 and Figure 10). The sample was left undisturbed for crystallization at room temperature. X-ray diffraction analysis of D5CuL2 (Figure 9) shows that the metal center of copper(II) is four-coordinated with a square planar geometry. Figure 10 shows that the Cu(II) ion has a five-coordinate square-based pyramid geometry with a t at 0.04. Selected bond distances and angle parameters for D5CuL2 and D6CuL2 are listed in Table 4.

The similarities include that the obtained crystals are that the four methoxy groups on the rotationally free phenyl rings point in opposite directions, which was also reported by Pucci [68]; and that the nitrate ion always acts as the counter ion, as confirmed by FTIR. It is probable that water or solvent molecules coordinate with the copper ion in solution and generate square-based pyramidal geometries because of the dynamic nature of the system. CCDC numbers of D5CuL2 and D6CuL2 complexes contain the supplementary crystallographic data presented in this paper. These data can be obtained free of charge via http://www.ccdc.cam.ac.uk/conts/retrieving.html (accessed on 28 April 2022) (from CCDC-2169382 and CCDC-2169383).

### 2.3. Biological Assays

It is important to generate new molecules to identify a candidate that can be used for the treatment of cancer in order to reduce/eliminate side effects, drug resistance and poor bioavailability. In vitro data obtained by proliferation assays provide useful initial information on the cytotoxicity of the agents in different cell lines.

#### Cytotoxicity Activity

The secondary ligands and coordination compounds were tested against SKLU-1 (human lung adenocarcinoma) and HeLa (human epithelial cervical carcinoma) cell lines. Table 5 shows the data of the inhibitory concentration 50 (CI_50_) values. Free ligands exhibit important cytotoxicity activity against the cell lines; however, the studied metal complexes show significantly higher activity against the same cell lines. These data suggest that generating copper coordination compounds improves cytotoxicity activity; this deportment has been studied in several mixed chelate complexes with curcumin [50], dimethoxycurcumin [39] and analogs [67], suggesting that solubility and stability in biological media must be the main factors. We propose that redox potential plays an important role.

In general, it is also observed that molecules that contain phenanthroline as a primary ligand are more active than coordination compounds that contain bipyridines. Additionally, compounds with substitutions with methyl groups at positions -5,6 are slightly more active than those with methyl groups at positions -4,7; this effect was observed by Chikira [68,69,70,71], who suggested that the effect could be due to interaction with DNA, as substituents in positions -4 or -7 decreased the association between the copper(II) compounds and DNA. On the other hand, it appears that the higher the molecular weight, the more active the coordination compounds; however, tetramethylated and diphenylated compounds are less active than dimethylated compounds; this phenomenon may be due to the fact that such molecules are more lipophilic and their crossing through the cell membrane is different, so activity decreases. Interestingly, in this work, the D1CuL1 compound did not show a cytotoxic effect against the tested HeLa cell lines; this led to further evaluations in different cancer cell lines to determine whether this molecule can present selectivity for lung-type cell lines.

In general, the copper(II) coordination compounds synthesized in this work show an improvement in biological activity compared to the first-generation Casiopeinas (Table 5) that contain the secondary ligand acetylacetonate (acac), which is the feedstock used to obtain the L1 and L2 heptanoids. This suggests that increasing the aliphatic chain and adding aromatic rings at the end of the chain improves this biological activity; however, third-generation Casiopeinas are less soluble in water. Previous reports mention that in first-generation Casiopeinas with different amino acids, the hydrophobicity of the ligands increases the cellular uptake of copper(II) in vitro [72]. Hydrophobic properties become more important, directly influencing biological activity, and are a descriptor in the transport of the drug to the site of action [73,74].

**Table 5:** Cytotoxicity assays for secondary ligands and complexes with L1 and L2 against SKLU-1 and HeLa cell lines.

Compound	IC_50_ SKLU-1 (μM)	IC_50_ HeLa (μM)	IC_50_ HeLa (μM) of First-Generation Casiopeinas
L1	52.56 ± 2.1	50.44 ± 2.3	-
D1CuL1	41.53 ± 0.5	N/A	42 ± 3.1 [25]
D2CuL1	14.36 ± 0.8	26.4 9 ± 0.7	41.7 ± 0.31 [75]
D3CuL1	8.90 ± 0.2	35.78 ± 0.7	-
D4CuL1	5.29 ± 0.9	7.61 ± 0.6	10.7 ± 0.9 [25]
D5CuL1	2.68 ± 0.2	1.91 ± 0.8	3.2 ± 0.03 [75]
D6CuL1	2.60 ± 0.5	2.21 ± 0.5	2.83 ± 0.09 [75]
D7CuL1	4.48 ± 1.5	4.48 ± 0.5	2.37 ± 0.4 [75]
D8CuL1	6.25 ± 0.9	6.74 ± 0.5	4.2 ± 0.6 [25]
L2	56.58 ± 1.8	109.05 ± 0.5	-
D1CuL2	21.82 ± 0.4	44.62 ± 1.1	42 ± 3.1 [25]
D2CuL2	9.49 ± 1.3	37.6 ± 1.9	41.7 ± 0.31 [75]
D3CuL2	6.84 ± 1.1	30.56 ± 2.5	-
D4CuL2	4.56 ± 0.6	13.26 ± 1.5	10.7 ± 0.9 [25]
D5CuL2	1.22 ± 0.1	2.01 ± 0.0005	3.2 ± 0.03 [75]
D6CuL2	1.19 ± 0.007	1.78 ± 0.1	2.83 ± 0.09 [75]
D7CuL2	2.68 ± 0.1	1.2 ± 0.05	2.37 ± 0.4 [75]
D8CuL2	2.38 ± 0.3	2.07 ± 0.3	4.2 ± 0.6 [25]
Cisplatin	9.56	5.1 ± 0.4 [25]	42 ± 3.1 [25]

### 2.4. Structure–Activity Relationships

Biological structure–activity relationships are a useful tool to describe or predict biological behavior according to structural, electronic or experimental parameters. These relationships can lead to the rational design of new molecules with optimal physicochemical or electronic properties to enhance biological activity. However, a challenge associated with systems with a metallic center is that most of the time, the descriptors do not work when the metal is changed. This occur for various reasons, such as the change in the geometries of the complexes or due to the lack of functionals that better describe this type of system.

**Figure 11:**
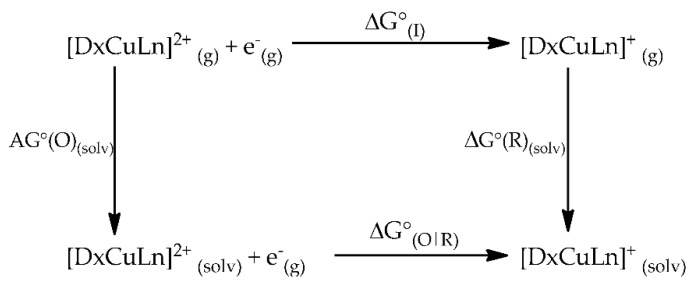
Born-Haber cycle used for the calculation of standard Gibbs free-energy change (ΔG°_O|R_) of mixed chelate copper(II) complexes.

It is necessary to identify parameters that elucidate whether there is a relationship between the structure/electronic properties and the cytotoxic activity of the copper(II) coordination compounds. The redox potential (E_1/2_), molar volume (V), ionization energy (IE) and electron affinity (EA) were obtained computationally from a single-point calculation based on optimized geometries and from single-point calculations of anionic and cationic molecules, respectively (Table 6). Complexes of copper(II) structures were constructed assuming a square-based pyramid geometry with the crystal structure of CasIII-ia compounds as a template [76]. Surprisingly, computational model was in agreement with the distances and bond angles with the X-ray structures obtained in this work (Figure 9 and Figure 10). On the other hand, the values obtained by the model (Figure 11) were close to the experimental values, so the procedure to determine the theoretical redox potential is valuable, as it can be used in cases in which experimental results are unavailable.

**Figure 12:**
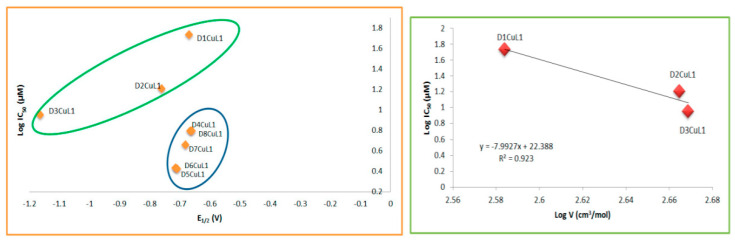
Relationship between molar volume or redox potential with antiproliferative activity of copper(II) coordination compounds with curcumin with CI_50_ in SKLU-1.

Although there is a large set of 16 complexes that have structural similarities, we decided to relate the parameters calculated for each family separately, that is, according to the secondary ligand. For the family of copper(II) coordination compounds with curcumin, the experimental redox potential (E_1/2_) and molar volume were found to be adequate to describe the antiproliferative activity in the SKLU-1 tumor cell line (Figure 12). We determined that the more negative the half-wave potential, the greater the cytotoxic activity. The molar volume implies a better antiproliferative effect only for the compounds with bipyridine ligands; this phenomenon was first observed for copper and ruthenium complexes [77]. The family of copper(II) coordination compounds with dimethoxycurcumin did not show a relationship with any descriptor for the SKLU and Hela cell lines.

**Figure 13:**
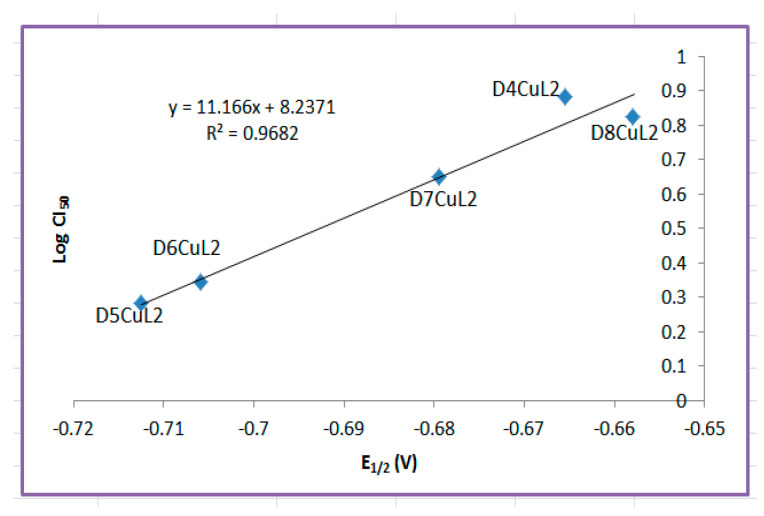
Relationship between redox potential and antiproliferative activity of copper(II) coordination compounds with curcumin with CI_50_ in HeLa.

A structure–activity relationship search was also performed using the IC_50_ values in HeLa cells for each family of compounds. For the family of copper(II) coordination compounds with curcumin, the experimental redox potential (E_1/2_) was found to be suitable to describe antiproliferative activity (Figure 13). The most important results from structure–activity relationships studies are that redox potential and molar volume can explain the antiproliferative activity of the family of complexes with curcumin, suggesting that its mechanism of action could be due to an increase in ROS generation, causing oxidative stress similar to behavior of first-generation of Casiopeinas [24]. On the other hand, it was observed that the family of copper(II) coordination compounds with dimethoxycurcumin shows no relationship with any parameter discussed here. This observation does not suggest that the mechanism of action of these molecules is not via redox but that there is no mathematical relationship between these parameters. On the contrary, although copper(II) coordination compounds with dimethoxycurcumin can carry out redox processes, a relationship with biological activity was not observed in HeLa and SKLU-1. This suggests that their mechanisms of action could be interaction with DNA, which is also a molecular target of the first generation of Casiopeinas.

## 3. Materials and Methods

All 1,10-phenantrolines and 2,2′-bipyridines with different substituents, copper(II) nitrate hemipentahydrate (Cu(NO_3_)_2_.5H_2_O), 2,4-pentanodione (C_5_H_8_O_2_), 4-hidroxy-3-methoxybenzaldehyde (C_8_H_8_O_3_), 3,4-dimethoxybenzaldehyde (C_9_H_10_O_3_), n-butylamine (C_4_H_11_N), tributyl borate (C_12_H_27_BO_3_), boric acid (H_3_BO_3_), potassium bromide (KBr), TBAPF_6_ (C_14_H_27_NPF_6_), trizma buffer and sulforhodamine B were acquired from Sigma-Aldrich, and solvents employed to synthesize (A.R. grade) were purchased from Quimica Alvi (Ciudad de Mexico, Mexico). All reagents were used directly without further purification. Penicillin-streptomycin and fetal bovine serum were purchased from GIBCO. DMEM-F12, trichloroacetic acid (TCA) and glacial acetic acid were purchased from J.T Baker.

### 3.1. Instrumentation

Elemental analyses of prepared compounds were performed with a Perkin-Elmer CHN 2400 elemental analyzer. Melting points (°C) were determined in capillary Pyrex using an SMP30 Stuart scientific instrument. Infrared spectra were analyzed on a Nicolet AVATAR 320 FT-IR spectrophotometer using KBr pellets in an interval range of 4000–400 cm^−1^. Magnetic moments of metal complexes were determined at room temperature on a magnetic susceptibility balance (Sherwood Scientific MKI, Cambridge UK) with the Gouy method. Ultraviolet-visible spectra (UV-vis) were obtained on a Hewlett Packard 5484 A UV-visible system diode array spectrophotometer in a range of 180 nm to 700 nm in dimethylsulfoxide (DMSO). Molar conductance of metal complexes was measured by a JENWAY 4330 conductivity-pH meter equilibrated at 25 °C using samples with final concentrations of 1 mM in DMSO. The spectra of EPR were measured in X-Band mode solid-state using a JEOL JES-TE300 spectrometer at a microwave frequency of 9.4 GHz and a center field of 300 mT at room temperature. The EPR spectra were simulated to obtain g_I_, g_II_ and hyperfine tensors using the EasySpin simulation package [78] for MATLAB R2019b. ^1^H and ^13^C NMR spectra of ligands were obtained on a Bruker Fourier 300 MHz and Bruker AVANCE III HD 700 MHz spectrometer using TMS (tetramethylsilane, Me_4_Si) as internal standard. The solvent used was DMSO-d^6^, which was acquired from Sigma-Aldrich. Mass spectra were recorded with a JEOL, SX 102 A spectrometer on a Bruker Microflex platform equipped with MALDI-flight time. Cyclic voltametric measurements were performed in DMSO using a PAR27 potentiostat/galvanostat with a conventional three-electrode array.

### 3.2. Syntheses of Ligands and Coordination Compounds

#### 3.2.1. Syntheses of Ligands Curcumin (L1) and Dimethoxycurcumin (L2)

Ligands were synthesized using known procedures describe by Pabon, with a modification introduced by Soria [37], which involves an aldol condensation reaction of 2,4-pentanodione with 4-hidroxy-3-methoxybenzaldehyde or 3,4-dimethoxybenzaldehyde to obtain curcumin (L1) and dimethoxycurcumin (L2), respectively (Figure 1). Pure 1,7-diarylheptanoid was obtained by column chromatography over silica gel (SiO_2_) using a hexane/EtOAc (30:70 *v*/*v*) mixture as the eluent. The pure compound was recrystallized in a methanol–acetone mixture. Ligands were characterized by elemental analysis, melting point, UV-vis spectrophotometry, NMR (^1^H and ^13^C) and mass spectrometry.

Curcumina (L1): bright orange solid (50% yield); C_21_H_20_O_6_ (368.37 g/mol); elemental analysis, %calculated (%found): C, 68.46 (68.78); H, 5.47 (5.49). m.p.: 174.6 °C; FT-IR (cm^−1^): 3438 ν(O-H), 1628 ν(C=O), 1510 ν(C=C), 2946 ν(C-H), 1280 ν(Ar-O); MALDI MS M^+^ (*m*/*z*): 368 (369); ^1^H NMR (DMSO-*d*_6,_ 300 MHz): ∂ 3.84 (s, 6H), 6.06 (s, 1H), 6.76 (d, 2H, *J* = 15.85 Hz), 6.83 (d, 2H, *J* = 8.18 Hz), 7.15 (dd, 2H, *J* = 1.95, 8.31 Hz), 7.32 (d, 2H, *J* = 1.97 Hz), 7.55 (d, 2H, *J* = 15.87 Hz), 9.67 (s, 2H), 16.41 (br s, 1H). ^13^C NMR (DMSO-d_6,_ 300 MHz): ∂ 56.15 (C-H), 56.09 (C-H), 101.30 (C-H), 111.82 (C_aryl_), 116.17 (C_aryl_), 121.55 (C_aryl_), 123.58 (C_vinyl_), 126.80 (C_aryl_), 141.16 (C_vinyl_), 148.45(C_aryl_), 149.81 (C_aryl_), 183.66 (C=O).

Dimethoxycurcumin (L2): orange solid (33% yield); C_23_H_24_O_6_ (396.43 g/mol); Elemental analysis, %calculated (%found): C, 69.68 (69.41); H, 6.1 (6.12). m.p.: 129.9 °C; FT-IR (cm^−1^): 3444 ν(O-H), 1622 ν(C=O), 1510 ν(C=C), 2917 ν(C-H), 1261 ν(Ar-O); MALDI MS M^+^ (*m*/*z*): 396 (397); ^1^H NMR (DMSO-*d*_6,_ 700 MHz): ∂ 3.80 (s, 6H), 3.83 (s, 6H), 6.11 (s, 1H), 6.83 (d, 2H, *J* = 16.73 Hz), 7.01 (d, 2H, J = 8.24 Hz), 7.26 (dd), 2H, *J* = 8.24 Hz), 7.35 (d, 2H), 7.59 (d, 2H, *J* = 15.78 Hz), 16.29 (br s, 1H). ^13^C NMR (DMSO-d_6,_ 700 MHz): ∂ 56.32 (C-H), 56.06 (C-H), 101.49 (C-H), 110.70 (C_aryl_), 112.29 (C_aryl_), 122.83 (C_vinyl_), 123.74 (C_aryl_), 128.09 (C_aryl_), 140.97(C_vinyl_), 149.51 (C_aryl_), 151.43 (C_aryl_), 183.69 (C=O).

#### 3.2.2. General Synthesis Procedure of Ternary Complexes of [Cu(Dn)(L1)]NO_3_ and [Cu(Dn)(L2)]NO_3_

In order to obtain mixed chelate copper(II) coordination compounds with L1 and L2 as secondary ligands, a general method was carried out following the methodology previously reported in [79,80,81]; however, a slight modification was made to raise reaction yields. Broadly, copper(II) nitrate hemipentahydrate (0.7 mmol, 1 M) was dissolved in 25 mL pure methanol solution, and diimine (0.7 mmol) was dissolved in 60 mL methanol solution. The latter solution was added dropwise to the copper(II) salt solution and stirred for 40 min. Then, after the addition, the previously deprotonated ligands, L1 or L2 (0.63 mmol, 0.232 g), were added. The reaction mixture was left for 5–6 h under continuous stirring with reflux. A change of color was observed. After cooling to room temperature, the formed solid was vacuum-filtered. Products were washed with EtOAc (3 × 20 mL) and cold water to remove residual reactants. The resulting precipitates were dried in a vacuum at room temperature. Between families of coordination compounds, the color of the precipitated powder depends on the presence of diimine.

**[Cu(D1)(L1)]NO_3_:** golden solid (98% yield); C_31_H_27_N_3_O_9_Cu (649.1 g/mol); elemental analysis, %calculated (%founded): C, 57.36 (57.13); H, 4.19 (4.17); N, 6.47 (6.46). m.p.: 248.0 °C; FT-IR (cm^−1^): 3423 ν(O-H), 1619 ν(C=N), 1598 ν(C-O), 1508 ν(C=C), 2969 ν(C-H), 1280 ν(Ar-O), 1384.7 ν (NO_3_), 480 ν (M-O); μ_eff_ = 1.05 B.M. Molar conductance Λ (DMSO) = 37.3 μS. MALDI MS M^+^ (*m*/*z*): 586.11 (586.9).

**[Cu(D2)(L1)]NO_3_:** orange solid (94% yield); C_33_H_31_N_3_O_9_Cu.H_2_O (695.17 g/mol); elemental analysis, %calculated (%founded): C, 57.01 (56.77); H, 4.78 (4.76); N, 6.04 (6.07). Decomposition temperature: 222.5 °C; FT-IR (cm^−1^): 3423 ν(O-H), 1621 ν(C=N), 1589 ν(C-O), 1508 ν(C=C), 2939 ν(C-H), 1280 ν(Ar-O), 1384.7 ν (NO_3_), 470 ν (M-O); μ_eff_ = 1.79 B.M. Molar conductance Λ (DMSO) = 33.03 μS. MALDI MS M^+^ (*m*/*z*): 614.47 (615.03).

**[Cu(D3)(L1)]NO_3_:** reddish solid (96% yield); C_33_H_31_N_3_O_9_Cu.H_2_O (695.17 g/mol); elemental analysis, %calculated (%founded): C, 57.01 (57.29); H, 4.78 (4.79); N, 6.04 (6.01). Decomposition temperature: 208.6 °C; FT-IR (cm^−1^): 3425 ν(O-H), 1600 ν(C=N), 1596 ν(C-O), 1510 ν(C=C), 2935 ν(C-H), 1280 ν(Ar-O), 1384.7 ν (NO_3_), 480 ν (M-O); μ_eff_ = 1.13 B.M. Molar conductance Λ (DMSO) = 32.9 μS. MALDI MS M^+^ (*m*/*z*): 614.47 (614.08).

**[Cu(D4)(L1)]NO_3_:** orange solid (94% yield); C_33_H_27_N_3_O_9_Cu (673.12 g/mol); elemental analysis, %calculated (%founded): C, 58.88 (58.63); H, 4.04 (4.02); N, 6.24 (6.28). Decomposition temperature: 249.2 °C; FT-IR (cm^−1^): 3436 ν(O-H), 1618 ν(C=N), 1589 ν(C-O), 1510 ν(C=C), 2944 ν(C-H), 1278 ν(Ar-O), 1384.7 ν(NO_3_), 476 ν(M-O); μ_eff_ = 1.10 B.M. Molar conductance Λ (DMSO) = 32.6 μS. MALDI MS M^+^ (*m*/*z*): 610.11 (610.98).

**[Cu(D5)(L1)]NO_3_:** reddish solid (80% yield); C_35_H_31_N_3_O_9_Cu.H_2_O (719.19 g/mol); elemental analysis, %calculated (%founded): C, 58.45 (58.70); H, 4.62 (4.59); N, 5.84 (5.82). Decomposition temperature: 200.5 °C; FT-IR (cm^−1^): 3419 ν(O-H), 1621 ν(C=N), 1596 ν(C-O), 1510 ν(C=C), 2977 ν(C-H), 1282 ν(Ar-O), 1384.7 ν(NO_3_), 478 ν(M-O); μ_eff_ = 1.35 B.M. Molar conductance Λ (DMSO) = 34.0 μS. MALDI MS M^+^ (*m*/*z*): 638.14 (638.04).

**[Cu(D6)(L1)]NO_3_:** orange solid (95% yield); C_35_H_31_N_3_O_9_Cu (701.18 g/mol); elemental analysis, %calculated (%founded): C, 59.95 (60.20); H, 4.45 (4.47); N, 5.99 (5.97). Decomposition temperature: 205.4 °C; FT-IR (cm^−1^): 3432 ν(O-H), 1621 ν(C=N), 1600 ν(C-O), 1511 ν(C=C), 2936 ν(C-H), 1278 ν(Ar-O), 1384.7 ν (NO_3_), 478 ν (M-O); μ_eff_ = 1.10 B.M. Molar conductance Λ (DMSO) = 59.1 μS. MALDI MS M^+^ (*m*/*z*): 638.14 (638.04).

**[Cu(D7)(L1)]NO_3_:** orange solid (95% yield); C_37_H_35_N_3_O_9_Cu.H_2_O (747.24 g/mol); elemental analysis, %calculated (%founded): C, 59.47 (59.20); H, 4.99 (4.97); N, 5.62 (5.59). Decomposition temperature: 243.2 °C; FT-IR (cm^−1^): 3446 ν(O-H), 1619 ν(C=N), 1594 ν(C-O), 1510 ν(C=C), 2925 ν(C-H), 1282 ν(Ar-O); 1384.7 ν(NO_3_), 478 ν(M-O); μ_eff_ = 1.92 B.M. Molar conductance Λ (DMSO) = 31.9 μS. MALDI MS M^+^ (*m*/*z*): 666.17 (667.18).

**[Cu(D8)(L1)]NO_3_:** orange solid (80% yield); C_45_H_35_N_3_O_9_Cu (825.32 g/mol); elemental analysis, %calculated (%founded): C, 65.48 (65.51); H, 4.27 (4.25); N, 5.09 (5.12). Decomposition temperature: 246.0 °C; FT-IR (cm^−1^): 3417 ν(O-H), 1618 ν(C=N), 1589 ν(C-O), 1510 ν(C=C), 2942 ν(C-H), 1280 ν(Ar-O); 1384 ν(NO^3^), 474 ν(M-O); μ_eff_ = 2.16 B.M. Molar conductance Λ (DMSO) = 32.8 μS. MALDI MS M^+^ (*m*/*z*): 762.17 (762.10).

**[Cu(D1)(L2)]NO_3_:** orange solid (88% yield); C_33_H_31_N_3_O_9_Cu (677.16 g/mol); elemental analysis, %calculated (%founded): C, 58.53 (58.24); H, 4.61 (4.59); N, 6.2 (6.18). m.p.: 236.9 °C; FT-IR (cm^−1^): 3427 ν(O-H), 1616 ν(C=N), 1579 ν(C-O), 1510 ν(C=C), 2948 ν(C-H), 1265 ν(Ar-O); 1384.7 ν(NO^3^), 486 ν(M-O); μ_eff_ = 1.91 B.M. Molar conductance Λ (DMSO) = 37.4 μS. MALDI MS M^+^ (*m*/*z*): 614.14 (615.30).

**[Cu(D2)(L2)]NO_3_:** orange solid (92% yield); C_35_H_35_N_3_O_9_Cu.H_2_O (723.22 g/mol); elemental analysis, %calculated (%founded): C, 58.12 (57.86); H, 5.15 (5.12); N, 5.81 (5.83). m.p.: 244.9 °C; FT-IR (cm^−1^): 3471 ν(O-H), 1616 ν(C=N), 1581 ν(C-O), 1510 ν(C=C), 2939 ν(C-H), 1265 ν(Ar-O); 1384.7 ν(NO_3_), 480 ν(M-O); μ_eff_ = 1.86 B.M. Molar conductance Λ (DMSO) = 23.2 μS. MALDI MS M^+^ (*m*/*z*): 642.17 (643.29).

**[Cu(D3)(L2)]NO_3_:** light green solid (84% yield); C_35_H_35_N_3_O_9_Cu.H_2_O (723.22 g/mol); elemental analysis, %calculated (%founded): C, 58.12 (58.39); H, 5.15 (5.18); N, 5.81 (5.84). m.p.: 262.7 °C; FT-IR (cm^−1^): 3452 ν(O-H), 1619 ν(C=N), 1581 ν(C-O), 1508 ν(C=C), 2939 ν(C-H), 1263 ν(Ar-O); 1384.7 ν(NO_3_), 484 ν(M-O); μ_eff_ = 1.9 B.M. Molar conductance Λ (DMSO) = 24.1 μS. MALDI MS M^+^ (*m*/*z*): 642.17 (642.10).

**[Cu(D4)(L2)]NO_3_:** light green solid (80% yield); C_35_H_31_N_3_O_9_Cu.2H_2_O (737.21 g/mol); elemental analysis, %calculated (%founded): C, 57.02 (56.75); H, 4.78 (4.76); N, 5.69 (5.66). m.p.: 254.3 °C; FT-IR (cm^−1^): 3436 ν(O-H), 1619 ν(C=N), 1581 ν(C-O), 1510 ν(C=C), 2939 ν(C-H), 1261 ν(Ar-O); 1384.7 ν(NO_3_), 462 ν(M-O); μ_eff_ = 1.9 B.M. Molar conductance Λ (DMSO) = 23.7 μS. MALDI MS M^+^ (*m*/*z*): 638.14 (638.03).

**[Cu(D5)(L2)]NO_3_:** solid (% yield); C_37_H_35_N_3_O_9_Cu (729.23 g/mol); elemental analysis, %calculated (%founded): C, 60.94 (60.68); H, 4.83 (4.82); N, 5.76 (5.74). Decomposition temperature: 244.5 °C; FT-IR (cm^−1^): 3428 ν(O-H), 1621 ν(C=N), 1579 ν(C-O), 1508 ν(C=C), 2935 ν(C-H), 1259 ν(Ar-O); 1384.7 ν(NO_3_), 472 ν(M-O); μ_eff_ = 2.26 B.M. Molar conductance Λ (DMSO) = 33.8 μS. MALDI MS M^+^ (*m*/*z*): 666.17 (668.24).

**[Cu(D6)(L2)]NO_3_:** solid (% yield); C_37_H_35_N_3_O_9_Cu (729.23 g/mol); elemental analysis, %calculated (%founded): C, 60.94 (61.19); H, 4.83 (4.85); N, 5.76 (5.79). Decomposition temperature: 237.0 °C; FT-IR (cm^−1^): 3444 ν(O-H), 1619 ν(C=N), 1581 ν(C-O), 1510 ν(C=C), 2935 ν(C-H), 1261 ν(Ar-O); 1384.7 ν(NO_3_), 472 ν(M-O); μ_eff_ = 1.96 B.M. Molar conductance Λ (DMSO) = 28.7 μS. MALDI MS M^+^ (*m*/*z*): 666.17 (668.24).

**[Cu(D7)(L2)]NO_3_:** solid (% yield); C_39_H_39_N_3_O_9_Cu (757.28 g/mol); elemental analysis, %calculated (%founded): C, 61.85 (61.56); H, 5.19 (5.21); N, 5.54 (5.51). Decomposition temperature: 241.0 °C; FT-IR (cm^−1^): 3446 ν(O-H), 1612 ν(C=N), 1579 ν(C-O), 1504 ν(C=C), 2931 ν(C-H), 1265 ν(Ar-O); 1384.7 ν(NO_3_), 484 ν(M-O); μ_eff_ = 2.10 B.M. Molar conductance Λ (DMSO) = 28.8 μS. MALDI MS M^+^ (*m*/*z*): 694.21 (696.14).

**[Cu(D8)(L2)]NO_3_:** solid (% yield); C_47_H_39_N_3_O_9_Cu (853.37 g/mol); elemental analysis, %calculated (%founded): C, 66.14 (65.86); H, 4.60 (4.63); N, 4.92 (4.94). Decomposition temperature: 238.8 °C; FT-IR (cm^−1^): 3413 ν(O-H), 1625 ν(C=N), 1562 ν(C-O), 1511 ν(C=C), 2933 ν(C-H), 1263 ν(Ar-O); 1384.7 ν(NO_3_), 480 ν(M-O); μ_eff_ = 2.30 B.M. Molar conductance Λ (DMSO) = 27.2 μS. MALDI MS M^+^ (*m*/*z*): 790.21 (791.56).

### 3.3. Cyclic Voltammetry Studies

Cyclic voltammetry was performed using a typical three-electrode cell comprising a pseudo-reference Ag, working glassy carbon electrodes and platinum wire as counter electrode. All electrochemical experiments were carried out using 1mM solutions of each coordination compound in DMSO in the presence of 0.1 M tetrabutylammonium hexafluorophosphate [NBu_4_][PF_6_] as supporting electrolyte. Before to each experiment, the working electrode was cleaned and polished with diamond powder (3 μm). All of the solutions were degassed with nitrogen prior to measurement. Cyclic voltammetry was initiated from open-circuit potential to the negative direction; the scan rate employed was 100 mVs^−1^ in both cathodic and anodic directions. On the other hand, by varying the scanning speed, the kinetics of the reactions can be studied, or the appearance of intermediate species can be detected accordingly in redox processes. We performed cyclic voltammetry with a variable scan rate between 20 and 1000 mV/s in the reductive direction. All potentials were reported versus the coupled ferrocene as internal standard (E°Fc^+^/Fc) according to IUPAC.

### 3.4. X-ray Crystallography

Single-crystal compounds D5CuL2 and D6CuL2 were coated with hydrocarbon oil (Parabar) for isolation, mounted on a glass fiber and placed on the goniometer head of the diffractometer for analysis. Suitable crystals were collected on a Bruker APEX II CCD diffractometer using Mo-Kα radiation (λ = 0.71073 Å) at 100 K from an Incoatec ImuS source and a Helios optic monochromator. The structures were solved using intrinsic phasing (SHELXT) and refined by full-matrix least-squares on F2 using the shelXle GUI. The crystallographic data and refinement details for the complexes reported in this paper are summarized in Table 7. Chemical structure drawings were produced using Mercury software.

### 3.5. Biological Assays

#### Cytotoxicity Activity

The cytotoxicity of curcumin (L1), dimethoxycurcumin (L2) and all complexes on tumor cells was determined using the protein-binding dye sulforhodamine B assay against two human cancer cell lines: SKLU-1 (non-small cell lung cancer) and HeLa (cell cervix cancer). The cell lines were cultured in supplemented DMEM-F12 medium (containing 10% fetal bovine serum, 1% non-essential amino acids and 1% penicillin-streptomycin). The plates were kept at 37 °C in a 5% CO_2_ atmosphere with 95% humidity. The viability of the cells used in the experiments was major at 95% as determined with trypan blue; after confluence, cells were used for all IC_50_ determinations. The next day, medium was withdrawn, and 90 μL of new medium and 10 μL of the individual complexes were added to each well. Test samples were dissolved in 1mL of DMSO to afford stock solutions (10 mM). After 24 h of treatment, the medium was retired, and plates were fixed by adding 100 μL trichloroacetic acid (TCA) and stored in a fridge for 60 min at 4 °C. The supernatant was scrapped, and the wells were washed thrice with water and air-dried. The fixed cells were stained with 50 μL of sulforhodamine B solution for 30 min in the dark; then, SRB solution was discarded, and plates were washed with 100 μL of acetic acid (1% *v*/*v*) and air-died. A volume of 50 μL of Tris was added to the plates to solubilize the SRB. Finally the plates were read at a wavelength of 570 nm. The results correspond to the average of 4 independent assays in triplicate for each combination. The concentration of complexes required for 50% inhibition of cell viability is commonly termed IC_50_ and calculated with a dose–response curve, which was plotted for each compound; the resulting concentration that resulted was estimated through fit sigmoidal analysis with Origin Pro 8 software.

### 3.6. Structure–ActivityRrelationships

#### 3.6.1. Computational Methods

Computational chemistry is a useful tool that can help to explain or predict the behavior and chemical reactivity of one or more molecules. We studied the redox potential behavior of these coordination compounds through computational calculations that involve obtaining physicochemical parameters such as Gibbs energies in the gas phase and with a solvation model. We calculated the redox potential of all families of coordination compounds of copper(II) (represents the oxidized complex) and its reduced species (coordination compounds of copper(I)) using Born–Haber cycles (Figure 11). DFT calculations were carried out to optimize geometries and determinate Gibbs energies of each compound with Gaussian 09.

#### 3.6.2. Computational Details

The crystal structure of copper(II) complexes were used as initial molecular geometries for optimization at the global hybrid functional M05-2x/LanL2DZ theoretical level with an SMD solvent model in which dimethylsulfoxide was employed as dissolvent to simulate the cyclic voltammetry experiment. Then, the obtained optimized molecules were used as a starting point for optimization of their reduced species (copper(I) complexes). Molecular geometries of copper(II/I) complexes were optimized in the gas phase at the same level of theory. The Gibbs free energy change from solvation theory calculations and gas phase calculations of each species was used to evaluate the standard Gibbs free energy change, ΔG°_O|R_ (Equation (1)):

(1) $$-\Delta G{{}^{\circ}}_{O|R}=\Delta G{{}^{\circ}}_{I}+\Delta \Delta G{{}^{\circ}}_{II}$$

where the Gibbs free energy change for the gas phase oxidation reaction is ΔG°*_I_* (Equation (2)), and the Gibbs free energy change of solvation is ΔG°*_II_* (Equation (3)).

(2) $$\Delta G{{}^{\circ}}_{I}=\Delta G{}^{\circ}{\left[\mathrm{CuDxLn}\right]}^{+}{}_{\left(g\right)}-\Delta G{}^{\circ}{\left[\mathrm{CuDxLn}\right]}^{2+}{}_{\left(g\right)}$$

(3) $$\Delta G{{}^{\circ}}_{II}=\Delta G{}^{\circ}{\left[\mathrm{CuDxLn}\right]}^{+}{}_{solv}-\Delta G{}^{\circ}{\left[\mathrm{CuDxLn}\right]}^{2+}{}_{solv}$$

We calculated the redox potential of a half reaction for a one-electron exchange using the Nernst equation (Equation (4)), where F is the Faraday constant. Here, experimental redox potentials are reported with respect to ferrocene/ferrocenium (E°F_c_^+^/F_c_) as internal standard reference. Therefore, the same methodology and theoretical level applied to copper complexes was used to calculate the Gibbs free energy change of ferrocene/ferrocenium, with a redox-coupled ΔG°_[FeCp2]_^0/+^ value of 5.3 eV at the same level of theory.

(4) $$E{{}^{\circ}}_{O|R}=-\frac{1}{F}\left(\Delta G{{}^{\circ}}_{O|R}-\Delta G{{}^{\circ}}_{{\left[\mathrm{FeCp}2\right]}^{0/+}}\right)$$

## 4. Conclusions

The ligands curcumin and dimethoxycurcumin were synthesized. For the latter, a good route for its purification was identified. Sixteen mixed chelate copper(II) coordination compounds were prepared (third generation of Casiopeinas) and characterized, showing a 1:1:1 stereochemistry (diimine:Cu:curcuminoid). These complexes can have a square-based pyramid or square planar geometry, depending on whether the water molecules are coordinated or not to the metal center according to X-ray diffraction. The synthesis of copper(II) complexes with curcumin and dimethoxycucumin can be used as a template for the generation of new molecules that present novel properties.

The measured cytotoxic activity of the coordination compounds against HeLa and SKLU-1 showed a improved biological activity compared to free ligands and that including a bioactive molecule as a secondary ligand in the Casiopeinas enhances their biological activity. One hypothesis to be resolved is that the toxicity of Casiopeinas may decrease due to the presence of these curcuminoids, given their low toxicity both in normal cells and in vivo, making them a potential alternative for the treatment of cancer.

It was found that the experimental redox potential (E_1/2_) is adequate to describe the antiproliferative activity in the SKLU-1 tumor cell line for copper(II) coordination compounds with curcumin, which was validated with theoretical models. Increased activity of coordination compounds is important in pharmaceuticals. Our results will allow us to generate important research around the most active molecules to guide them towards pharmaceutical applications.

## Figures and Tables

**Table 1:** UV-vis spectral band assignments and molar extinction coefficient (ε) of ligands and copper complexes.

Compound	λ_max_(nm) π-π*	ε (L/mol cm)	λ_max_(nm) TCLM	ε (L/mol cm)	λ_max_(nm) TCML	ε (L/mol cm)
D1CuL1	294	31,451	430	64,287	454	62,853
D2CuL1	292	22,776	430	42,317	454	41,947
D3CuL1	304	28,563	426	36,928	448	33,804
D4CuL1	266	48,706	430	57,630	454	56,402
D5CuL1	268	51,897	430	59,041	454	56,908
D6CuL1	276	42,400	428	35,698	452	32,659
D7CuL1	274	34,157	432	33,142	456	32,775
D8CuL1	282	53,109	430	42,733	454	40,475
D1CuL2	313	22,461	430	24,832	458	23,216
D2CuL2	310	25,161	430	23,350	458	21,809
D3CuL2	321	17,513	430	20,741	458	19,205
D4CuL2	275	42,754	433	33,220	458	30,807
D5CuL2	276	52,001	433	34,631	458	32,386
D6CuL2	284	44,217	433	33,226	458	31,128
D7CuL2	282	49,471	433	32,936	458	30,671
D8CuL2	290	53,569	436	11,448	460	10,582

**Table 2:** Mass spectral data (*m*/*z*) of ligands and copper(II) complexes.

Compound	Molecular Weight (g/mol)	Molecular Ion (Theoretical *m*/*z*)	Molecular Ion (Obtained *m*/*z*)
Curcumin (L1)	368.37	368	369.2
[Cu(2,2′-bipyridine)(curcumina)]NO_3_ (D1CuL1)	649.01	586.1	586.9
[Cu(4,4′-dimethyl-2,2′-bipyridine)(curcumin)]NO_3_ (D2CuL1)	695.17	614.4	615.0
[Cu(5,5′-dimethyl-2,2′-bipyridine)(curcumin)]NO_3_ (D3CuL1)	695.17	614.4	614.0
[Cu(1,10-phenanthroline)(curcumin)]NO_3_ (D4CuL1)	673.12	610.1	610.9
[Cu(4,7-dimethyl-1,10-phenanthroline)(curcumin)]NO_3_ (D5CuL1)	719.19	638.1	638.0
[Cu(5,6-dimetyl-1,10-phenanthroline)(curcumin)]NO_3_ (D6CuL1)	701.18	638.1	638.0
[Cu(3,4,7,8-tetrametyl-1,10-phenanthroline)(curcumin)]NO_3_ (D7CuL1)	747.24	666.1	667.1
[Cu(4,7-diphenyl-1,10-phenanthroline)(curcumin)]NO_3_ (D8CuL1)	825.32	762.1	762.1
Dimethoxycurcumin (L2)	396.43	396.4	397.7
[Cu(2,2′-bipyridine)(dimethoxycurcumin)]NO_3_ (D1CuL2)	677.16	614.1	615.3
[Cu(4,4′-dimethyl-2,2′-bipyridine)(dimethoxycurcumin)]NO_3_ (D2CuL2)	723.22	642.1	643.2
[Cu(5,5′-dimethyl-2,2′-bipyridine)(dimethoxycurcumin)]NO_3_ (D3CuL2)	723.22	642.1	642.1
[Cu(1,10-phenanthroline)(dimethoxycurcumin)]NO_3_ (D4CuL2)	737.21	638.1	638.0
[Cu(4,7-dimethyl-1,10-phenanthroline)(dimethoxycurcumin)]NO_3_ (D5CuL2)	729.23	666.1	668.2
[Cu(5,6-dimethyl-1,10-phenanthroline)(dimethoxycurcumin)]NO_3_ (D6CuL2)	729.23	666.1	668.2
[Cu(3,4,7,8-tetramethyl-1,10-phenanthroline)(dimethoxycurcumin)]NO_3_ (D7CuL2)	757.28	694.2	696.1
[Cu(4,7-diphenyl-1,10-phenanthroline)(dimethoxycurcumin)]NO_3_ (D8CuL2)	853.37	790.2	791.5

**Table 3:** Summary of cyclic voltametric parameters for copper(II) coordination compounds.

Compound	Epc (V)	Epa (V)	ΔE (V)	E_1/2_ (V) (Fc^+^/Fc)
L1	−0.978	0.488	1.467	---
D1CuL1	−0.429	0.130	0.560	−0.668
D2CuL1	−0.484	0.023	0.507	−0.758
D3CuL1	−0.474	0.067	0.542	−1.164
D4CuL1	−0.407	0.140	0.547	−0.665
D5CuL1	−0.440	0.123	0.563	−0.712
D6CuL1	−0.611	−0.04	0.571	−0.705
D7CuL1	−0.405	0.154	0.560	−0.679
D8CuL1	−0.491	0.126	0.617	−0.657
L2	−1.05	---	---	---
D1CuL2	−0.444	0.139	0.623	−0.656
D2CuL2	−0.469	0.117	0.586	−0.709
D3CuL2	−0.390	0.162	0.552	−0.656
D4CuL2	−0.373	0.168	0.541	−0.656
D5CuL2	−0.447	0.088	0.535	−0.701
D6CuL2	−0.388	0.158	0.546	−0.682
D7CuL2	−0.396	0.096	0.492	−0.690
D8CuL2	−0.171	0.122	0.293	−0.559

**Table 4:** Selected bond lengths (Å) and angles (°) for compounds D5CuL2 and D6CuL2.

Compound	Bond Atom-Atom (A°)	Lengths	Bond Atom-Atom (A°)	Angles (°)
D5CuL2 square planar geometry	Cu-N1	1.977	O1-Cu-O2	94.68
	Cu-N2	1.993	N1-Cu-N2	81.84
	Cu-O1	1.906	O1-Cu-N1	92.37
	Cu-O2	1.886	O2-Cu-N2	89.45
	Cu-O (H_2_O)	5.456	O1-Cu-N2	168.51
	Cu-O (NO_3_)	6.582	O2-Cu-N1	166.09
D6CuL2 square-based pyramid geometry	Cu-N1	2.013	O1-Cu-O2	94.68
	Cu-N2	2.022	N1-Cu-N2	81.84
	Cu-O1	1.921	O1-Cu-N1	92.37
	Cu-O2	1.911	O2-Cu-N2	89.45
	Cu-O (H_2_O)	2.260	O1-Cu-N2	168.51
	Cu-O (NO_3_)	4.958	O2-Cu-N1	166.09

**Table 6:** Molar volume (cm^3^/mol), ionization energy (IE), electron affinities (EA) and theoretical redox potential (E_1/2_) for complexes under study.

Compound	Molar Volume (cm^3^/mol)	IE (eV)	EA (eV)	E_1/2_ (V)
D1CuL1	383.712	8.04749134	2.82789556	−0.878171
D2CuL1	462.274	7.99361656	2.76906755	−0.930038
D3CuL1	466.413	8.00534368	2.7998987	−0.872582
D4CuL1	410.031	8.10950385	2.87669331	−0.725702
D5CuL1	437.24	8.04221051	2.80906527	−0.749246
D6CuL1	417.529	8.07647188	2.85005565	−0.849173
D7CuL1	373.335	8.01205011	2.79094547	−0.907115
D8CuL1	492.824	8.07950481	2.86419938	−0.873203
D1CuL2	451.839	8.07429826	2.84794598	−0.836537
D2CuL2	450.172	8.01993014	2.78888151	−0.858569
D3CuL2	386.932	8.03231631	2.82004192	−0.840938
D4CuL2	393.699	8.13593635	2.8959002	−0.728105
D5CuL2	422.974	8.06659075	2.82811977	−0.809915
D6CuL2	466.821	8.10139715	2.86922906	−0.782915
D7CuL2	545.631	8.03569891	2.8086884	−0.799709
D8CuL2	536.71	8.09949401	2.87311289	−0.452801

**Table 7:** Crystallographic data and structural refinement parameters for D5CuL2 and D6CuL2.

Collection Parameters	D5CuL2	D6CuL2
Empirical formula	C_37_H_35_N_3_O_9_Cu	C_37_H_35_N_3_O_9_Cu
Formula weight	729.23 g/mol	729.23 g/mol
Temperature (K)	100	100
Wavelength (Ǻ)	1.5417	0.71073
Crystal system	Triclinic	Triclinic
Space group	P-1	P-1
a (Ǻ)	7.7004 (7)	10.0604 (7)
b (Ǻ)	13.7026 (12)	13.6229 (9)
c (Ǻ)	17.1812 (15)	14.0129 (9)
α (°)	110.132 (5)	70.5193 (12)
β(°)	94.503 (6)	76.4453 (13)
γ(°)	91.987 (6)	71.7320 (13)
Volume (Ǻ^3^)	1693.2 (3)	1701.1 (2)
Z	2	1
D_calc_ (mg/m^3^)	1.466	1.485
Absorption coefficient (mm^−1^)	1.462	0.709
F(000)	778	793
Crystal size (mm^3^)	0.300 × 0.041 × 0.036	0.150 × 0.092 × 0.056
Theta range for data collection (°)	2.752 to 68.243	1.558 to 27.442
Index ranges	−9 ≤ h ≤ 9, −16 ≤ k ≤ 16, −20 ≤ 1 ≤ 20	−13 ≤ h ≤ 13, −17 ≤ k ≤ 17, −18 ≤ l ≤ 18
Reflections collected	45,131	27,234
Independent reflections	5817 [R(int) = 0.0466]	7773 [R(int) = 0.0363]
Refinement method	Full-matrix least-squares on F^2^	Full-matrix least-squares on F^2^
Data/restraints/parameters	5818/563/584	7773/360/582
Goodness-of-fit on F2	1.086	1.040
Final R indices (I > 2sigma(I))	R1 = 0.0538, wR2 = 0.1580	R1 = 0.0355, wR2 = 0.0889
R indices (all data)	R1 =0.0588, wR2 = 0.1637	R1 = 0.0477, wR2 = 0.0889
Largest diff. peak and hole e Ǻ^−3^	0.878 and −0.651	0.366 and −0.520
Extinction coefficient	n/a	n/a

## Supplementary Materials

The following are available online at: https://www.mdpi.com/article/10.3390/molecules27113504/s1, CCDC-2169382 and CCDC-2169383 contain the supplementary crystallographic data for this paper. These data can be obtained free of charge via http://www.ccdc.cam.ac.uk/conts/retrieving.html (accessed on 28 April 2022).

## References

[1] Guzmán-Méndez O., González F., Bernès S., Flores-Álamo M., Ordóñez-Hernández J., García-Ortega H., Guerrero J., Qian W., Aliaga-Alcalde N., Gasque L. (2018). Coumarin Derivative Directly Coordinated to Lanthanides Acts as an Excellent Antenna for UV–Vis and Near-IR Emission. Inorg. Chem..

[2] Farrell N. (1989). Transition Metal Complexes as Drugs and Chemotherapeutic Agents, Met. Complexes as Drugs Chemother. Agents.

[3] Annaraj J., Srinivasan S., Ponvel K., Athappan P. (2005). Mixed ligand copper(II) complexes of phenanthroline/bipyridyl and curcumin diketimines as DNA intercalators and their electrochemical behavior under Nafion^®^ and clay modified electrodes. J. Inorg. Biochem..

[4] Akimenko N., Cheltsov P., Balcarová Z., Kleinwächter V., Yevdokimov Y.U. (1985). A study of interactions of platinum (II) compounds with DNA by means of CD spectra of solutions and liquid crystalline microphases of DNA. Gen. Physiol. Biophys..

[5] Sharma K., Chandra S., Basu D. (1987). Synthesis and antiarthritic study of a new orally active diferuloyl methane (curcumin) gold complex. Inorg. Chim. Acta.

[6] Manikandan P., Anandan R., Nagini S. (2009). Evaluation of *Azadirachta indica* Leaf Fractions for in Vitro Antioxidant Potential and Protective Effects against H_2_O_2_-Induced Oxidative Damage to pBR322 DNA and Red Blood Cells. J. Agric. Food Chem..

[7] Barik A., Mishra B., Shen L., Mohan H., Kadam R., Dutta S., Zhang H.-Y., Priyadarsini K.I. (2005). Evaluation of a new copper(II)–curcumin complex as superoxide dismutase mimic and its free radical reactions. Free Radic. Biol. Med..

[8] Huang W., Wei W., Shen Z. (2014). Drug-like chelating agents: A potential lead for Alzheimer’s disease. RSC Adv..

[9] Binolfi A., Lamberto G.R., Duran R., Quintanar L., Bertoncini C.W., Souza J.M., Cerveñansky C., Zweckstetter M., Griesinger C., Fernández C.O. (2008). Site-Specific Interactions of Cu(II) with α and β-Synuclein: Bridging the Molecular Gap between Metal Binding and Aggregation. J. Am. Chem. Soc..

[10] Santini C., Pellei M., Gandin V., Porchia M., Tisato F., Marzano C. (2013). Advances in Copper Complexes as Anticancer Agents. Chem. Rev..

[11] WHO (2020). Cancer Today. https://gco.iarc.fr/today/online-analysis-table?v=2020&mode=cancer&mode_population=continents&population=900&populations=900&key=asr&sex=0&cancer=39&type=1&statistic=5&prevalence=0&population_group=0&ages_group%5B%5D=0&ages_group%5B%5D=17&group_cancer=1&include_nmsc=1&include_nmsc_other=1.

[12] Alderden R.A., Hall M.D., Hambley T. (2006). The Discovery and Development of Cisplatin. J. Chem. Educ..

[13] Guo Z., Sadler P.J. (1999). Metals in Medicine. Angew. Chem. Int. Ed..

[14] Solans X., Ruiz-Ramírez L., Gasque L., Briansó J.L. (1987). Structure of (1,10-phenanthroline)(salicylaldehydato)copper(II) nitrate. Acta Crystallogr. Sect. C Cryst. Struct. Commun..

[15] Solans X., Ruiz-Ramírez L., Martinez A., Gasque L., Briansó J.L. (1988). Structures of chloro(glycinato)(1,10-phenanthroline)copper(II) monohydrate (I) and aqua(1,10-phenanthroline)(L-phenylalaninato)copper(II) nitrate monohydrate (II). Acta Crystallogr. Sect. C Cryst. Struct. Commun..

[16] Carvallo-Chaigneau F., Trejo-Solís C., Gómez-Ruiz C., Rodríguez-Aguilera E., Macías-Rosales L., Cortés-Barberena E., Cedillo-Peláez C., Gracia-Mora I., Ruiz-Azuara L., Madrid-Marina V. (2007). Constantino-Casas, Casiopeina III-ia induces apoptosis in HCT-15 cells in vitro through caspase-dependent mechanisms and has antitumor effect in vivo. BioMetals.

[17] Trejo-Solís C., Palencia G., Zuñiga S., Rodríguez-Ropon A., Osorio-Rico L., Luvia S.T., Gracia-Mora I., Marquez-Rosado L., Sánchez A., Moreno-García M.E. (2005). Sotelo, Cas Ilgly Induces Apoptosis in Glioma C6 Cells In Vitro and In Vivo through Caspase-Dependent and Caspase-Independent Mechanisms. Neoplasia.

[18] Serment-Guerrero J., Cano-Sanchez P., Reyes-Perez E., Velazquez-Garcia F., Bravo-Gomez M., Ruiz-Azuara L. (2011). Genotoxicity of the copper antineoplastic coordination complexes casiopeinas^®^. Toxicol. Vitr..

[19] Figueroa-DePaz Y., Resendiz-Acevedo K., Dávila-Manzanilla S.G., García-Ramos J.C., Ortiz-Frade L., Serment-Guerrero J., Ruiz-Azuara L. (2022). DNA, a target of mixed chelate copper(II) compounds (Casiopeinas^®^) studied by electrophoresis, UV–vis and circular dichroism techniques. J. Inorg. Biochem..

[20] Gutiérrez A.G., Vázquez-Aguirre A., García-Ramos J.C., Flores-Alamo M., Hernández-Lemus E., Ruiz-Azuara L., Mejía C. (2013). Copper(II) mixed chelate compounds induce apoptosis through reactive oxygen species in neuroblastoma cell line CHP-212. J. Inorg. Biochem..

[21] Hernández-Esquivel L., Marín-Hernández A., Pavón N., Carvajal K., Moreno-Sánchez R. (2006). Cardiotoxicity of copper-based antineoplastic drugs casiopeinas is related to inhibition of energy metabolism. Toxicol. Appl. Pharmacol..

[22] Ramirez-Palma L.G., Espinoza-Guillen A., Nieto-camacho F., López-Guerra A., Gómez-Vidales V., Cortés-Guzmán F., Ruiz-Azuara L. (2021). Intermediate Detection in the Casiopeina—Cysteine Interaction Ending in the Disulfide Bond Formation and Copper Reduction. Molecules.

[23] Marín-Hernández A., Gracia-Mora I., Ruiz-Ramírez L., Moreno-Sánchez R. (2003). Toxic effects of copper-based antineoplastic drugs (Casiopeinas^®^) on mitochondrial functions. Biochem. Pharmacol..

[24] Kachadourian R., Brechbuhl H.M., Ruiz-Azuara L., Gracia-Mora I., Day B.J. (2010). Casiopeína IIgly-induced oxidative stress and mitochondrial dysfunction in human lung cancer A549 and H157 cells. Toxicology.

[25] Bravo-Gómez M.E., García-Ramos J.C., Gracia-Mora I., Ruiz-Azuara L. (2009). Antiproliferative activity and QSAR study of copper(II) mixed chelate [Cu(N–N)(acetylacetonato)]NO3 and [Cu(N–N)(glycinato)]NO3 complexes, (Casiopeínas^®^). J. Inorg. Biochem..

[26] Stanić Z. (2016). Curcumin, a Compound from Natural Sources, a True Scientific Challenge—A Review. Plant Foods Hum. Nut..

[27] Epstein J., Sanderson I.R., MacDonald T.T. (2010). Curcumin as a Therapeutic Agent: The Evidence from in vitro, Animal and Human Studies. Br. J. Nutr..

[28] Nelson K.M., Dahlin J.L., Bisson J., Graham J., Pauli G.F., Walters M.A. (2017). The essential medicinal chemistry of curcumin. J. Med. Chem..

[29] Aggarwal B.B., Surh Y.J., Shishodia S. (2007). The Molecular Targets and Therapeutic Uses of Curcumin in Health and Disease.

[30] Kunnumakkara A.B., Bordoloi D., Padmavathi G., Monisha J., Roy N.K., Prasad S., Aggarwal B.B. (2017). Curcumin, the golden nutraceutical: Multitargeting for multiple chronic diseases. Br. J. Pharmacol..

[31] Cheng A.L., Hsu C.-H., Lin J.K., Hsu M.M., Ho Y.-F., Shen T.S., Ko J.Y., Lin J.T., Lin B.-R., Ming-Shiang W. (2001). Phase I clinical trial of curcumin, a chemopreventive agent, in patients with high-risk or pre-malignant lesions. Anticancer Res..

[32] Pabon H.J.J. (1964). Synthesis of curcumin and related compounds. Recueil.

[33] Leung M.H.M., Harada T., Kee T.W. (2013). Delivery of Curcumin and Medicinal Effects of the Copper(II)-Curcumin Complexes. Curr. Pharm. Des..

[34] Sharma R.A., Steward W.P., Gescher A.J. (2007). Pharmacokinetics and Pharmacodynamics of Curcumin. Mol. Targets Ther. Uses Curcumin Health Dis..

[35] Anand P., Kunnumakkara A.B., Newman R.A., Aggarwal B.B. (2007). Newman, Bioavailability of Curcumin: Problems and Promises reviews Bioavailability of Curcumin: Problems and Promises. Mol. Pharm..

[36] Schneider C., Gordon O.N., Edwards R.L., Luis P.B. (2015). Degradation of Curcumin: From Mechanism to Biological Implications. J. Agric. Food Chem..

[37] Kunwar A., Simon E., Singh U., Chittela R.K., Sharma D., Sandur S.K., Priyadarsini I.K. (2011). Interaction of a curcumin analogue dimethoxycurcumin with DNA. Chem. Biol. Drug Des..

[38] Meza-Morales W., Estévez-Carmona M.M., Alvarez-Ricardo Y., Obregón-Mendoza M.A., Cassani J., Ramírez-Apan M.T., Escobedo-Martínez C., Soriano-García M., Reynolds W.F., Enríquez R.G. (2019). Full Structural Characterization of Homoleptic Complexes of Diacetylcurcumin with Mg, Zn, Cu, and Mn: Cisplatin-level Cytotoxicity in Vitro with Minimal Acute Toxicity in Vivo. Molecules.

[39] Shahabadi N., Falsafi M., Moghadam N.H. (2013). DNA interaction studies of a novel Cu(II) complex as an intercalator containing curcumin and bathophenanthroline ligands. J. Photochem. Photobiol. B Biol..

[40] Banerjee R. (2014). Inhibitory Effect of Curcumin-Cu(II) and Curcumin-Zn(II) Complexes on Amyloid-Beta Peptide Fibrillation. Bioinorg. Chem. Appl..

[41] Mary C.P.V., Vijayakumar S., Shankar R. (2018). Metal chelating ability and antioxidant properties of Curcumin-metal complexes—A DFT approach. J. Mol. Graph. Model..

[42] Correa-Ascencio M., Galván-Miranda E.K., Rascón-Cruz F., Jiménez-Sandoval O., Jiménez-Sandoval S.J., Cea-Olivares R., Jancik V., Toscano R.A., García-Montalvo V. (2010). Lanthanide(III) Complexes with 4,5-Bis(diphenylphosphinoyl)-1,2,3-triazolate and the Use of 1,10-Phenanthroline As Auxiliary Ligand. Inorg. Chem..

[43] Refat M.S. (2013). Synthesis and characterization of ligational behavior of curcumin drug towards some transition metal ions: Chelation effect on their thermal stability and biological activity. Spectrochim. Acta Part A Mol. Biomol. Spectrosc..

[44] Pröhl M., Schubert U., Weigand W., Gottschaldt M. (2015). Metal complexes of curcumin and curcumin derivatives for molecular imaging and anticancer therapy. Coord. Chem. Rev..

[45] Rigamonti L., Orteca G., Asti M., Basile V., Imbriano C., Saladini M., Ferrari E. (2018). New curcumin-derived ligands and their affinity towards Ga^3+^, Fe^3+^ and Cu^2+^: Spectroscopic studies on complex formation and stability in solution. N. J. Chem..

[46] Priyadharshini N., Iyyam P.S., Subramanian S., Venkatesh P. (2015). Venkatesh, Synthesis, spectroscopic characterization and DNA interaction of schiff base curcumin Cu(II), Ni(II) and Zn(II) complexes. Pharma Chem..

[47] Pi Z., Wang J., Jiang B., Cheng G., Zhou S. (2015). A curcumin-based TPA four-branched copper(II) complex probe for in vivo early tumor detection. Mater. Sci. Eng. C.

[48] Zhou S., Xue X., Jiang B., Tian Y. (2011). Metal complexes of a novel bis-β-diketone-type ligand and its copper(II) complexes of two-photon biological imaging. Sci. China Chem..

[49] Xue X., Wang J., Si G., Wang C., Zhou S. (2016). Synthesis, DNA-binding properties and cytotoxicity evaluation of two copper(II) complexes based on curcumin. Transit. Met. Chem..

[50] Wang J., Wei D., Jiang B., Liu T., Ni J., Zhou S. (2014). Two copper(II) complexes of curcumin derivatives: Synthesis, crystal structure and in vitro antitumor activity. Transit. Met. Chem..

[51] JPorkodi J., Raman N. (2017). Synthesis, characterization and biological screening studies of mixed ligand complexes using flavonoids as precursors. Appl. Organomet. Chem..

[52] Boorman P.M., Greenwood N.N. (1968). and Hildon, M.A. Some diphosohine complexes of tungsten-(III) and –(IV). J. Chem. Soc. A.

[53] Geary W. (1971). The use of conductivity measurements in organic solvents for the characterisation of coordination compounds. Co-ord. Chem. Rev..

[54] Ismail E.H., Sabry D.Y., Mahdy H., Khalil M.M.H. (2014). Synthesis and Characterization of some Ternary Metal Complexes of Curcumin with 1,10-phenanthroline and their Anticancer Applications. J. Sci. Res..

[55] Goswami T.K., Gadadhar S., Gole B., Karande A.A., Chakravarty A.R. (2013). Photocytotoxicity of copper(II) complexes of curcumin and N-ferrocenylmethyl-l-amino acids. Eur. J. Med. Chem..

[56] Deepthi T.V., Venugopalan P. (2016). Synthesis, DNA-binding, and cytotoxic studies on three copper(II) complexes of unsymmetrical synthetic analogues of curcumin. J. Coord. Chem..

[57] Banerjee S., Chakravarty A.R. (2015). Metal Complexes of Curcumin for Cellular Imaging, Targeting, and Photoinduced Anticancer Activity. Acc. Chem. Res..

[58] Joseph J., Suman A., Nagashri K., Joseyphus R.S., Balakrishnan N. (2017). Synthesis, characterization and biological studies of copper(II) complexes with 2-aminobenzimidazole derivatives. J. Mol. Struct..

[59] Bain G.A., Berry J.F. (2008). Diamagnetic Corrections and Pascal’s Constants. J. Chem. Educ..

[60] Garribba E., Micera G. (2006). The Determination of the Geometry of Cu(II) Complexes: An EPR Spectroscopy Experiment. J. Chem. Educ..

[61] García-Giménez J.L., González-Álvarez M., Liu-González M., Macías B., Borrás J., Alzuet G. (2009). The development of metal-based synthetic nucleases: DNA binding and oxidative DNA cleavage of a mixed copper(II) complex with N-(9H-purin-6-yl)benzenesulfonamide and 1,10-phenantroline. Antitumor activity in human Caco-2 cells and Jurkat T lymphocy. J. Inorg. Biochem..

[62] Raman N., Sobha S. (2010). Synthesis, characterization, DNA interaction and antimicrobial screening of isatin-based polypyridyl mixed ligand Cu(II) and Zn(II) complexes. J. Serbian Chem. Soc..

[63] Masek A., Chrzescijanska E., Zaborski M. (2013). Characteristics of curcumin using cyclic voltammetry, UV–vis, fluorescence and thermogravimetric analysis. Electrochim. Acta.

[64] Călinescu M., Fiastru M., Bala D., Mihailciuc C., Negreanu-Pîrjol T., Jurca B. (2019). Synthesis, characterization, electrochemical behavior and antioxidant activity of new copper(II) coordination compounds with curcumin derivatives. J. Saudi Chem. Soc..

[65] Zhu J., Sanidad K.Z., Sukamtoh E., Zhang G. (2017). Potential roles of chemical degradation in the biological activities of curcumin. Food Funct..

[66] Meza-Morales W., Machado-Rodriguez J.C., Alvarez-Ricardo Y., Obregón-Mendoza M.A., Nieto-Camacho A., Toscano R.A., Soriano-García M., Cassani J., Enríquez R.G. (2019). A New Family of Homoleptic Copper Complexes of Curcuminoids: Synthesis, Characterization and Biological Properties. Molecules.

[67] Wanninger S., Lorenz V., Subhan A., Edelmann F.T. (2015). Metal complexes of curcumin—Synthetic strategies, structures and medicinal applications. Chem. Soc. Rev..

[68] Pucci D., Bellini T., Crispini A., D’Agnano I., Liguori P.F., Garcia-Orduña P., Pirillo S., Valentini A., Zanchetta G. (2012). DNA binding and cytotoxicity of fluorescent curcumin-based Zn(ii) complexes. MedChemComm.

[69] Aliaga-Alcalde N., Marqués-Gallego P., Kraaijkamp M., Herranz-Lancho C., Dulk H.D., Görner H., Roubeau O., Teat S.J., Weyhermüller T., Reedijk J. (2010). Copper Curcuminoids Containing Anthracene Groups: Fluorescent Molecules with Cytotoxic Activity. Inorg. Chem..

[70] Chikira M., Tomizawa Y., Fukita D., Sugizaki T., Sugawara N., Yamazaki T., Sasano A., Shindo H., Palaniandavar M., Antholine W.E. (2002). DNA-fiber EPR study of the orientation of Cu(II) complexes of 1,10-phenanthroline and its derivatives bound to DNA: Mono(phenanthroline)-copper(II) and its ternary complexes with amino acids. J. Inorg. Biochem..

[71] Hirohama T., Kuranuki Y., Ebina E., Sugizaki T., Arii H., Chikira M., Selvi P.T., Palaniandavar M. (2005). Copper(II) complexes of 1,10-phenanthroline-derived ligands: Studies on DNA binding properties and nuclease activity. J. Inorg. Biochem..

[72] Bravo-gómez M.E., Dávila-manzanilla S., Flood-garibay J., Muciño M. Á., Mendoza Á., García-ramos J.C., Moreno-esparza R., Ruiz-azuara L. (2012). Secondary Ligand Effects on the Cytotoxicity of Several Casiopeína’s Group II Compounds. J. Mex. Chem. Soc..

[73] Yilmaz V.T., Icsel C., Suyunova F., Aygun M., Aztopal N., Ulukaya E. (2016). Ni(ii)/Cu(ii)/Zn(ii) 5,5-diethylbarbiturate complexes with 1,10-phenanthroline and 2,2′-dipyridylamine: Synthesis, structures, DNA/BSA binding, nuclease activity, molecular docking, cellular uptake, cytotoxicity and the mode of cell death. Dalton Trans..

[74] Moghassemi S., Hadjizadeh A. (2014). Nano-niosomes as nanoscale drug delivery systems: An illustrated review. J. Control. Release.

[75] Davila-Manzanilla S.G., Figueroa-De-Paz Y., Mejia C., Ruiz-Azuara L. (2017). Synergistic effects between a copper-based metal Casiopeína III-ia and cisplatin. Eur. J. Med. Chem..

[76] Tovar-Tovar A., Ruiz-Ramírez L., Campero A., Romerosa A., Moreno-Esparza R., Rosales-Hoz M.J. (2004). Structural and reactivity studies on 4,4-dimethyl-2,2-bipyridine acetylacetonate copper(II) nitrate (CASIOPEINA III-ia) with methionine, by UV-visible and EPR techniques. J. Inorg. Biochem..

[77] Reina M., Hernández-Ayala L.F., Bravo-Gómez M.E., Gómez V., Ruiz-Azuara L. (2020). Second generation of Casiopeinas^®^: A joint experimental and theoretical study. Inorganica Chim. Acta.

[78] Stoll S., Schweiger A. (2006). EasySpin, a comprehensive software package for spectral simulation and analysis in EPR. J. Magn. Reson..

[79] Ruiz-Azuara L. (1997). United States Patent.

[80] Ruiz-Azuara L. (1996). United States Patent.

[81] Ruiz-Azuara L. (1997). United States Patent.

