# Supplementary material for: Casiopeinas of Third Generations: Synthesis, Characterization, Cytotoxic Activity and Structure–Activity Relationships of Mixed Chelate Compounds with Bioactive Secondary Ligands
Source: Molecules. 2022 May 30;27(11):3504. doi: 10.3390/molecules27113504 (PMC9182210; doi:10.3390/molecules27113504)
Supplement: Supplementary file 1 [file molecules-27-03504-s001.zip › molecules-1728319-Supplementary.pdf]

Supplementary Information

Casiopeinas of Third Generations: Synthesis, Characterization, Cytotoxic Activity and Structure-Activity Relationships of Mixed Chelate Compounds with Bioactive Secondary Ligands.

*Yeshenia Figueroa-DePaz, Jaime Pérez-Villanueva, Olivia Soria-Arteche, Diego Martínez-Otero, Virginia Gómez-Vidales, Luis Ortiz-Frade and Lena Ruiz-Azuara**

Correspondence: lenar701@gmail.com

CONTENTS

1H AND 13C NUCLEAR MAGNETIC RESONANCE

MASS SPECTROMETRY

VOLTAMPEROMETRY CYCLIC

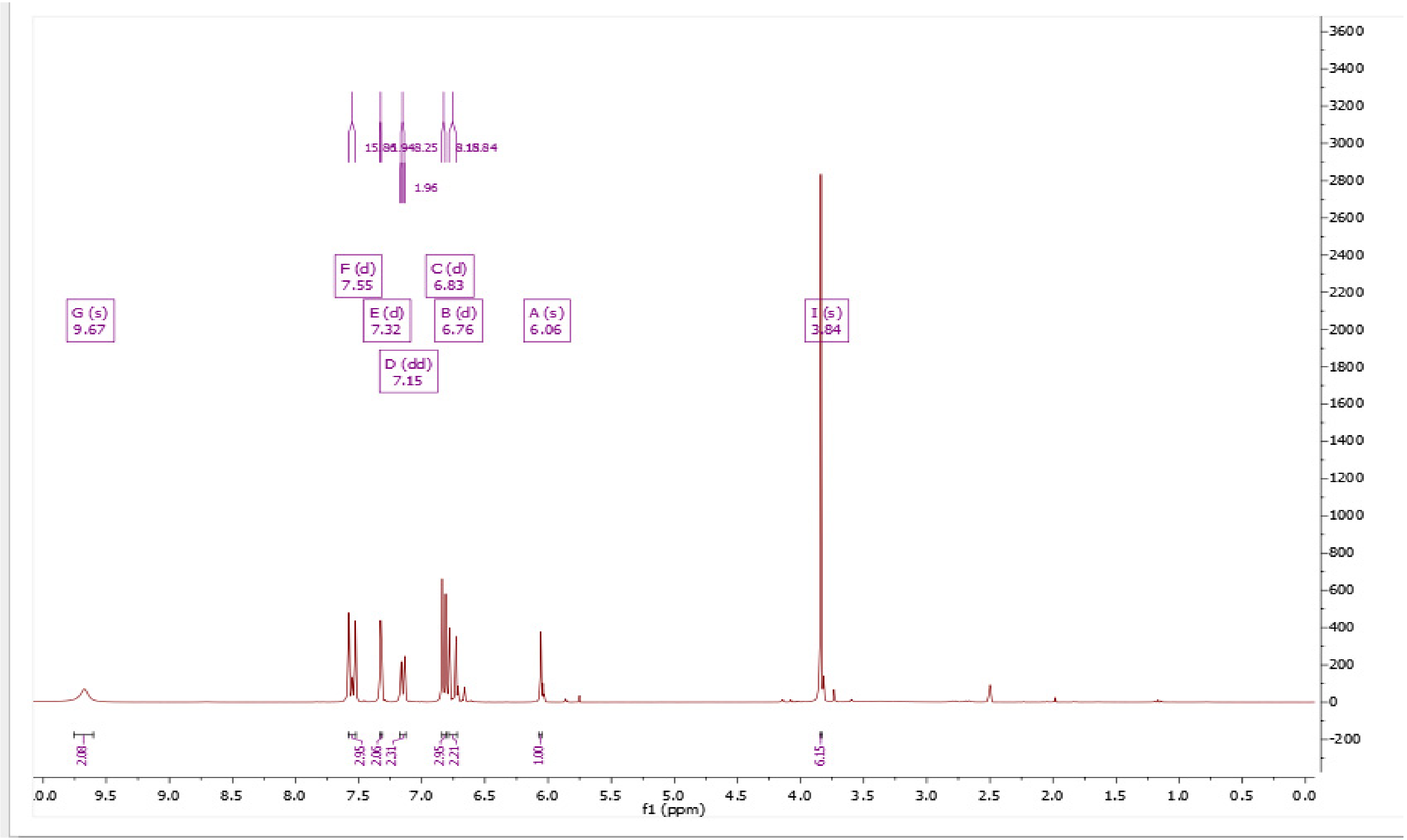

Figure S1. 300 MHz ^{1}H NMR spectrum of curcumin.

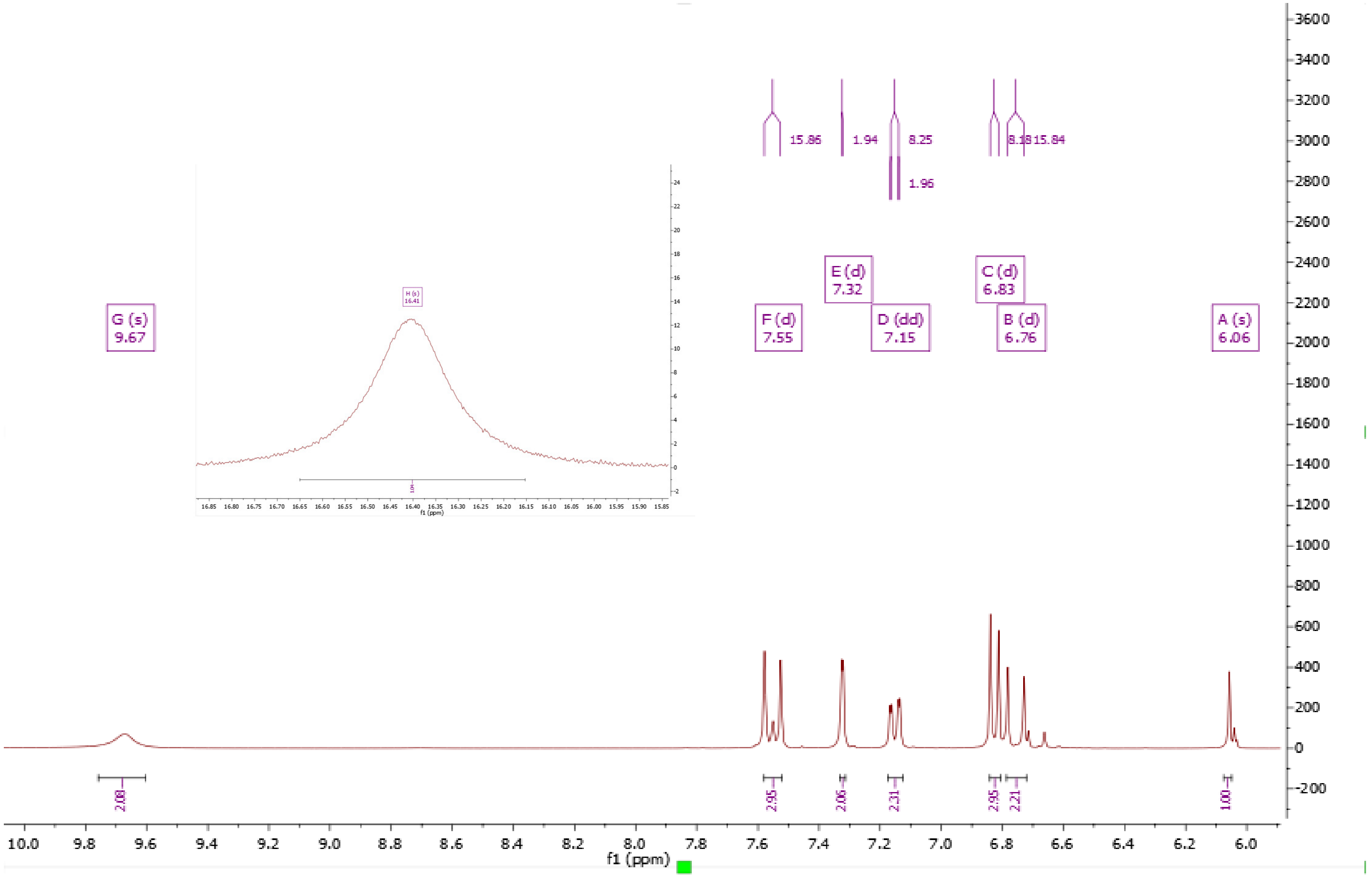

Figure S2. 300 MHz ^{1}H NMR spectrum expansion of curcumin.

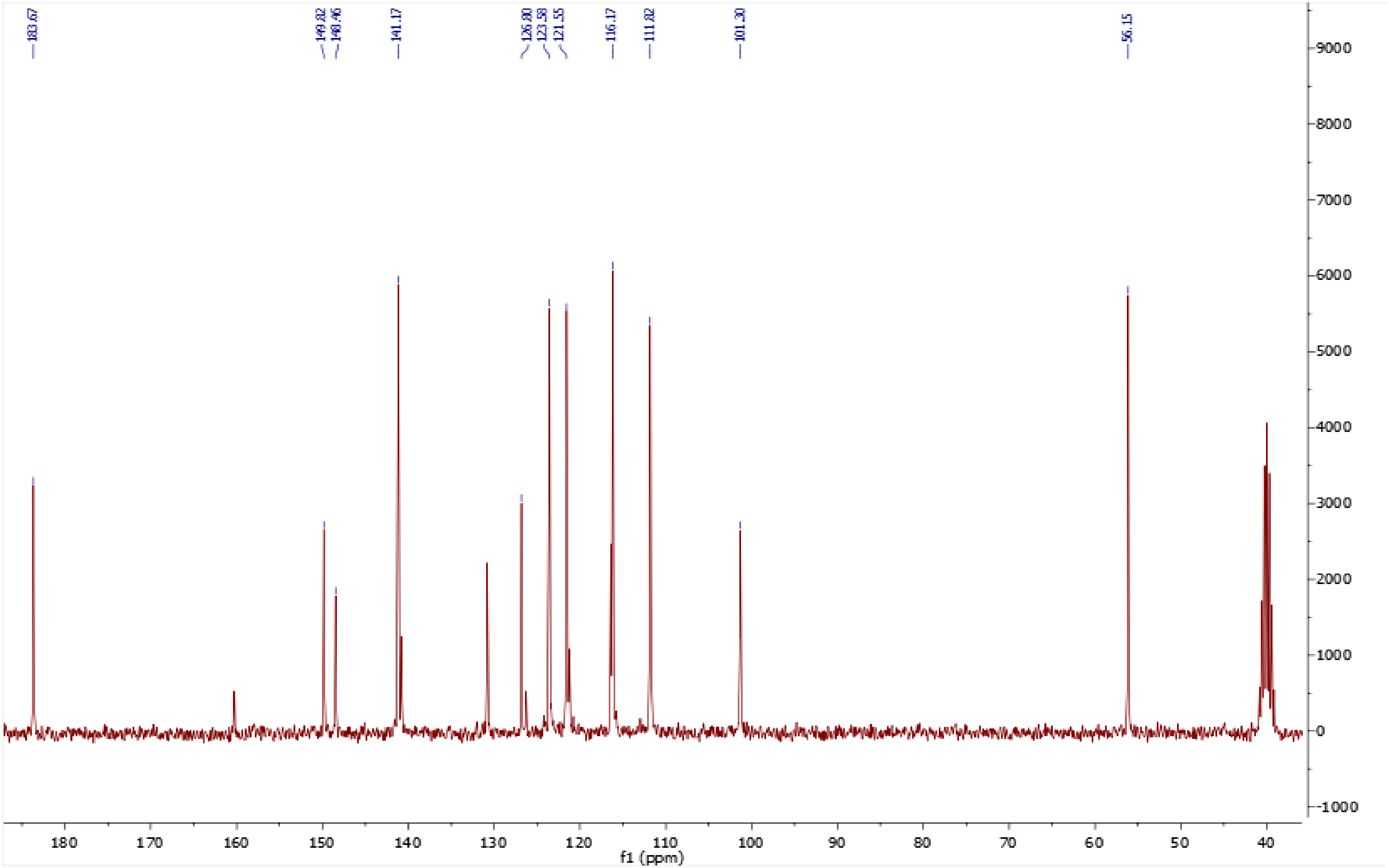

Figure S3. 300 MHz ^{13}C NMR spectrum of curcumin.

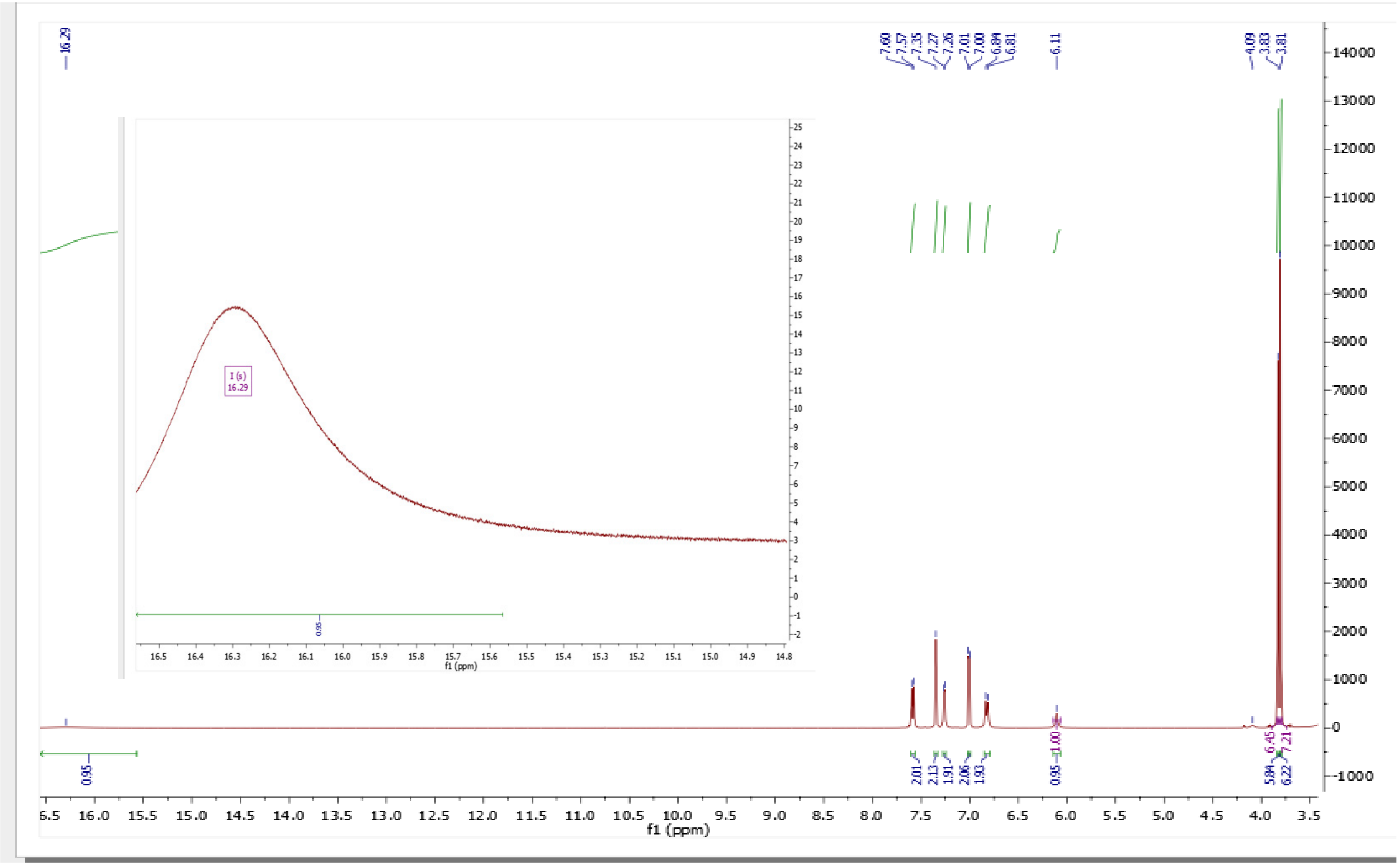

Figure S4. 700 MHz ^{1}H NMR spectrum of dimethoxycurcumin.

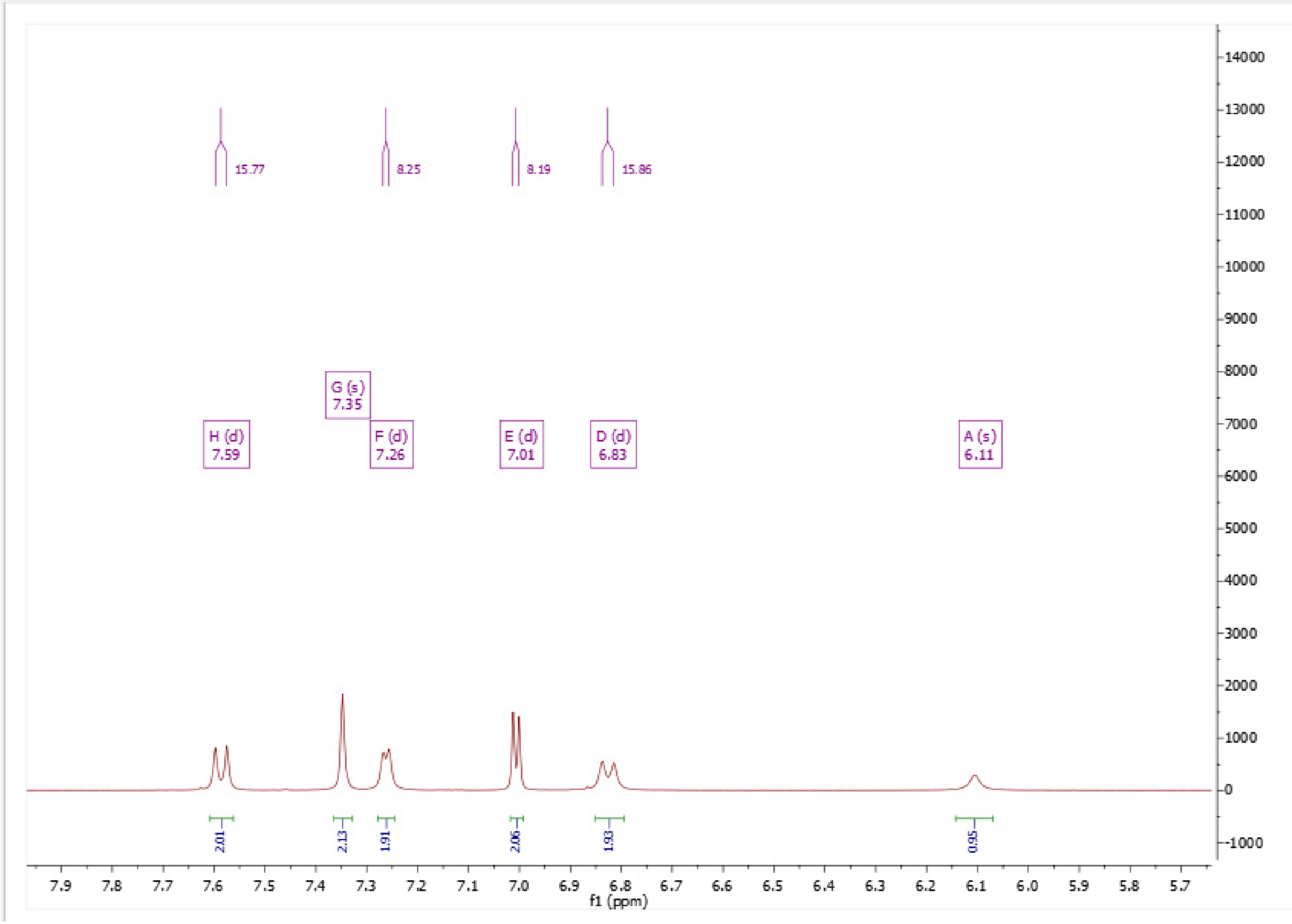

Figure S5. 700 MHz ^{1}H NMR spectrum expansion of dimethoxycurcumin.

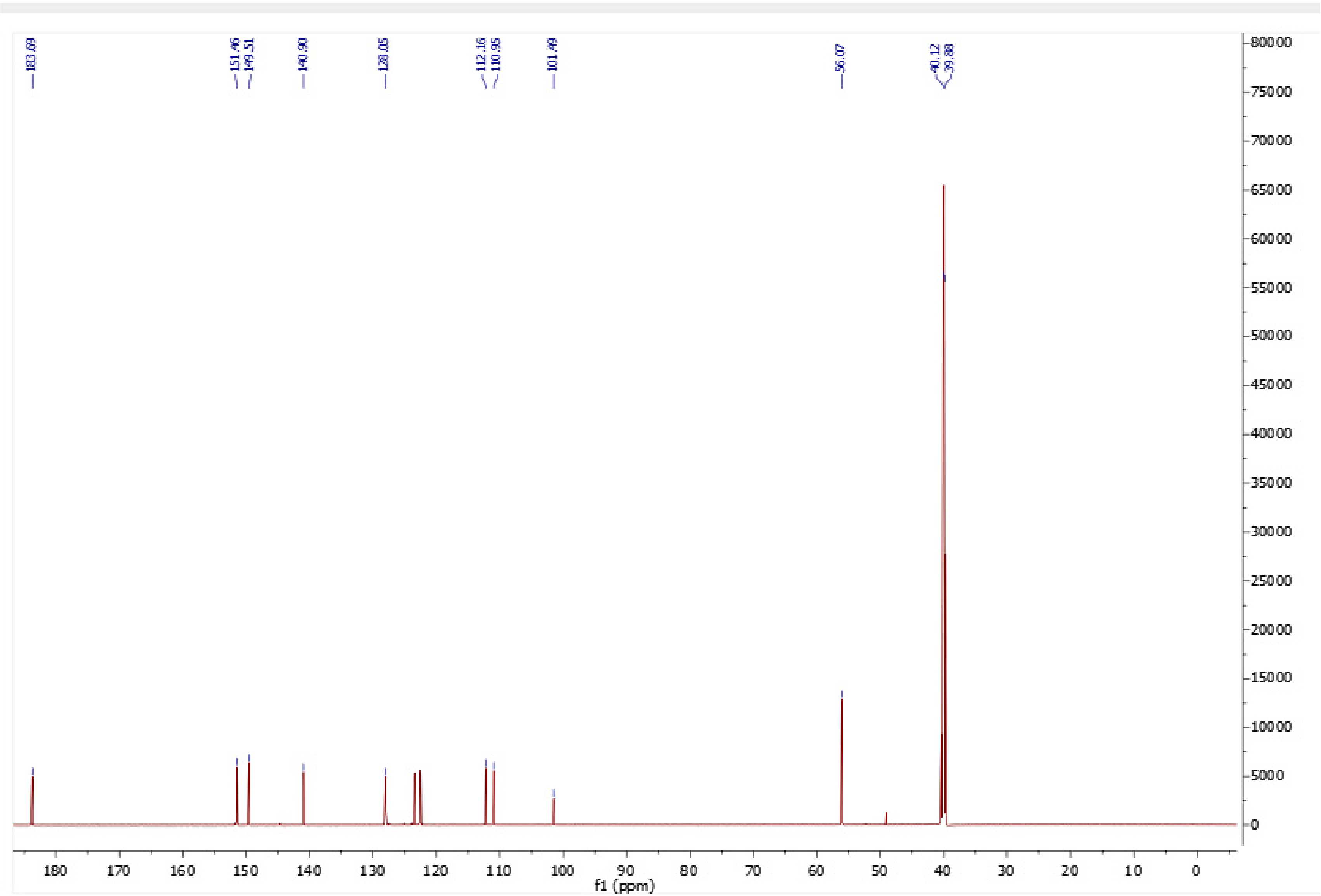

Figure S6. 700 MHz ^{13}C NMR spectrum of dimethoxycurcumin.

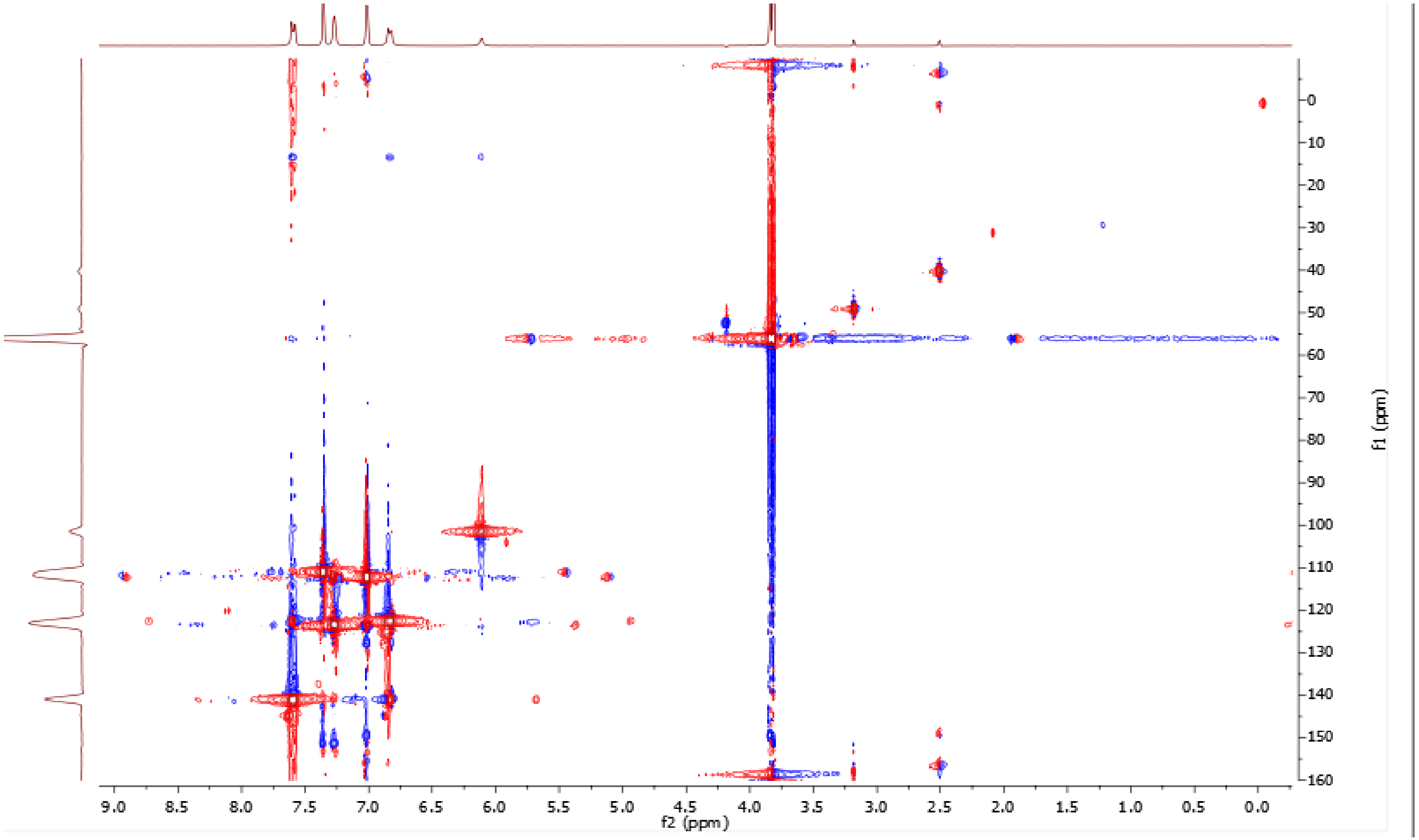

Figure S7. 700 MHz HSQC NMR spectrum of dimethoxycurcumin.

Table S1. EPR parameters of the Cu(II) complexes.

**Compounds**	$g_{\perp}$	$g_{\parallel}$	$A_{\perp}$ x10^{-4} cm^{-1}	$A_{\parallel}$ x10^{-4}/cm^{-1}	gII/AII
D1CuL1	2.07	2.22	3.46	111.09	200.10
D2CuL1	2.08	2.15	2.24	220.67	97.70
D3CuL1	2.07	2.20	0.33	28.83	765.61
D4CuL1	2.07	2.23	8.99	109.00	205.32
D5CuL1	2.07	2.21	2.47	95.67	231.53
D6CuL1	2.09	2.21	0.98	15.62	1408.15
D7CuL1	2.07	2.21	2.34	25.75	860.58
D8CuL1	2.07	2.22	3.61	40.49	549.27
D1CuL2	2.04	2.20	2.21	138.89	158.75
D2CuL2	2.05	2.22	0.45	3.33	6666.00
D3CuL2	2.04	2.24	3.79	171.19	131.37
D4CuL2	---	---	---	---	---
D5CuL2	2.06	2.13	89.03	231.33	92.23
D6CuL2	2.05	2.25	0.19	179.07	126.04
D7CuL2	2.04	2.31	10.01	128.79	179.43
D8CuL2	2.04	2.22	40.26	280.27	79.53

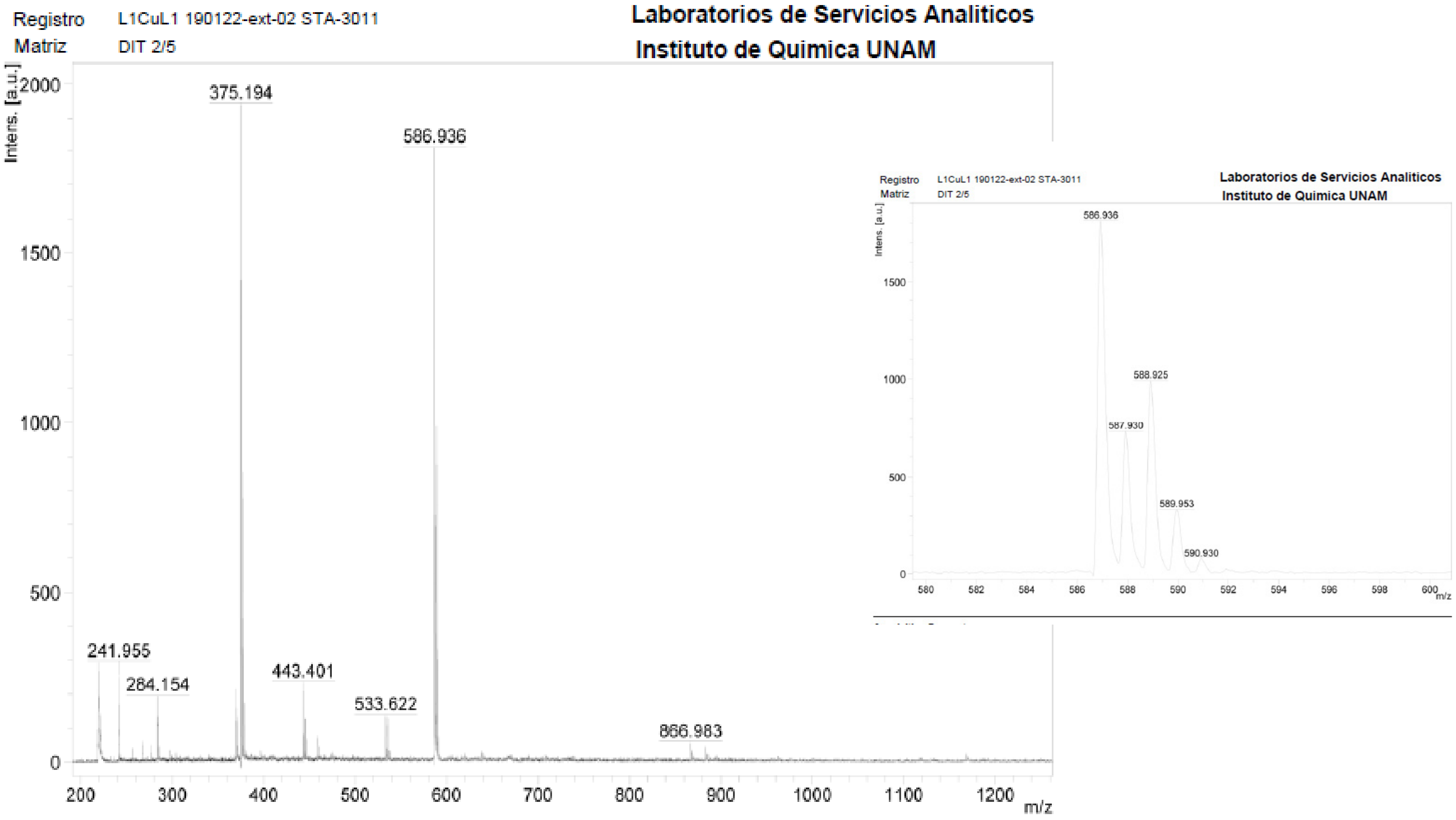

Figure S8. Mass Spectrum D1CuL1.

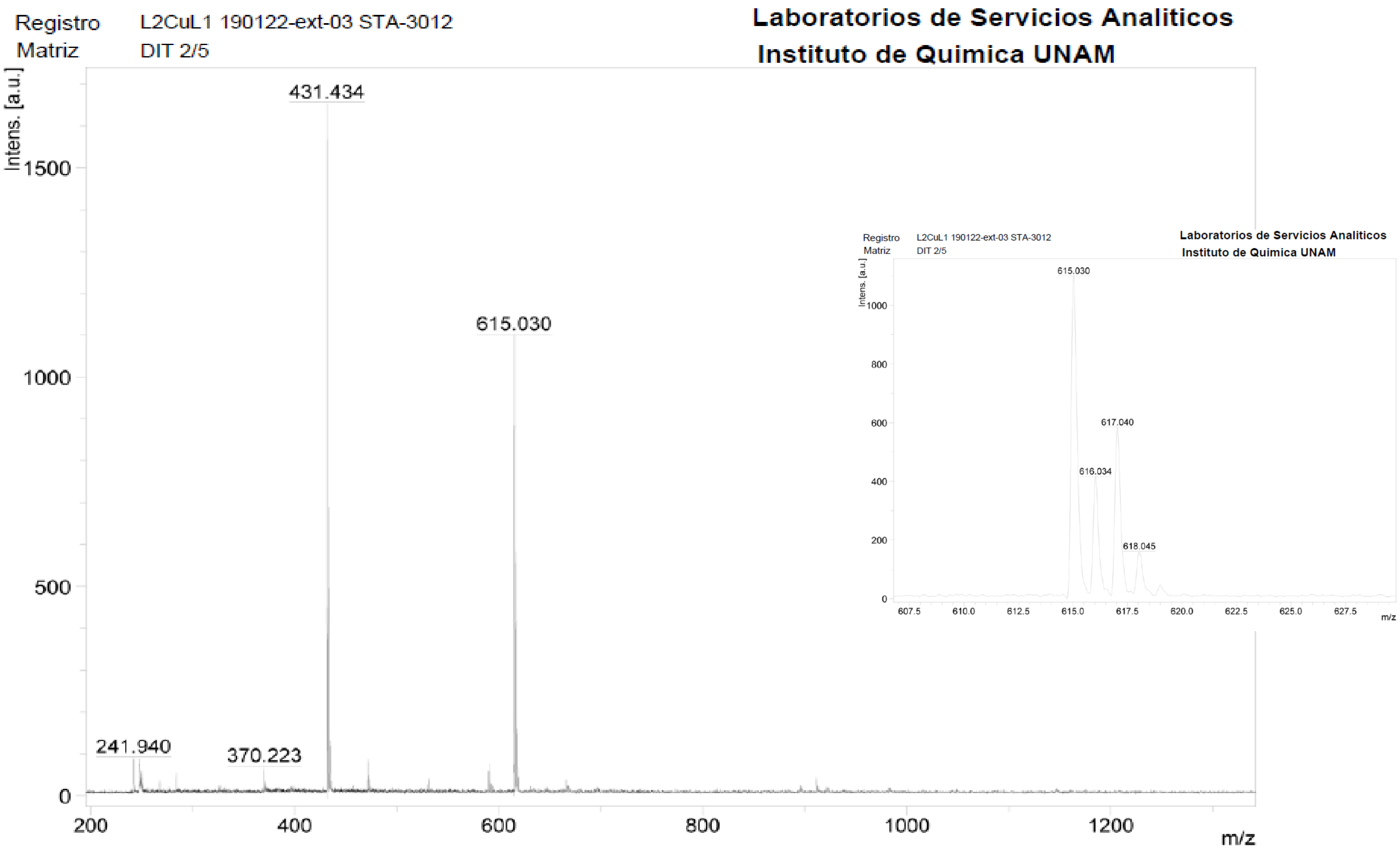

Figure S9. Mass Spectrum D2CuL1.

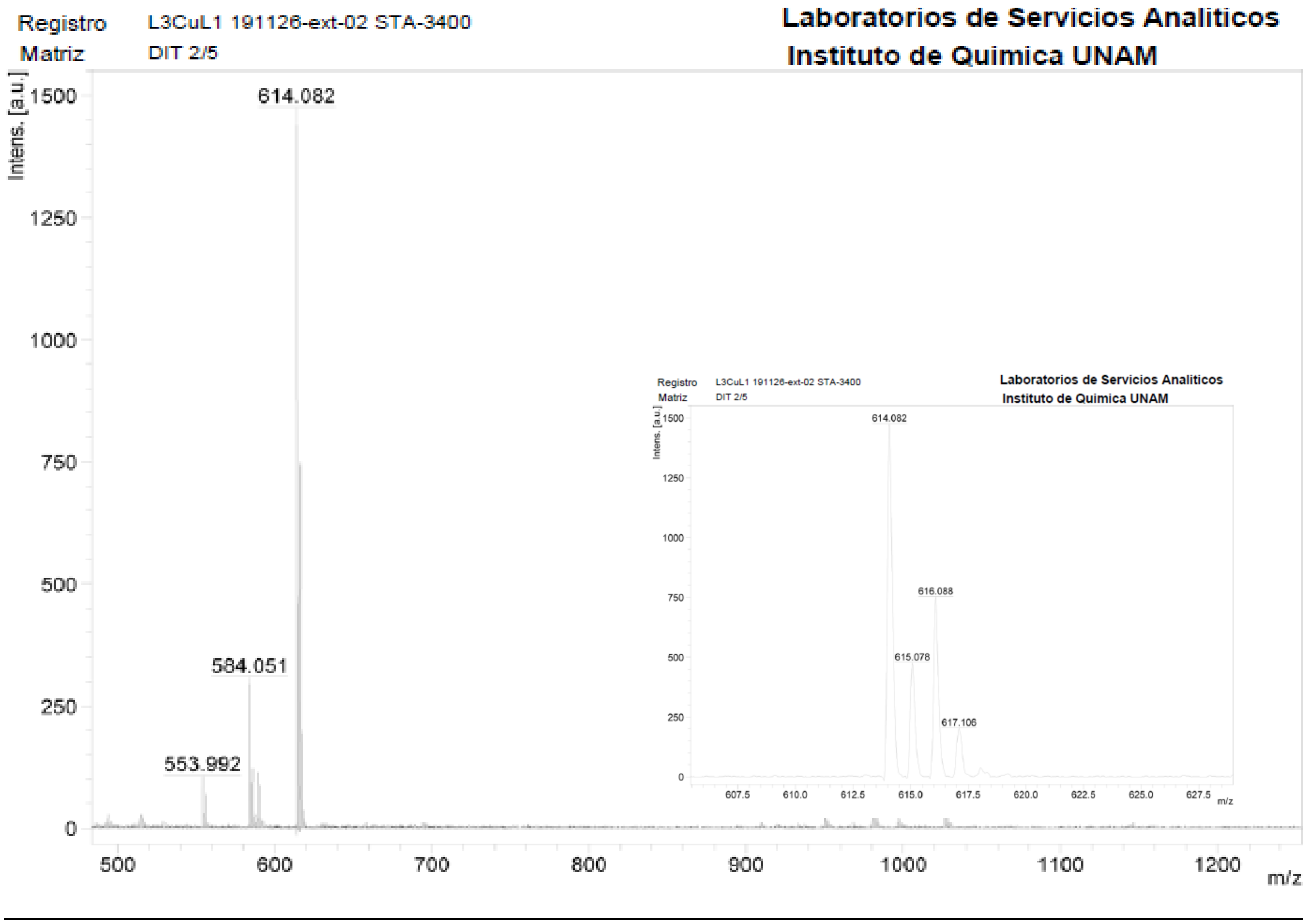

Figure S10. Mass Spectrum D3CuL1.

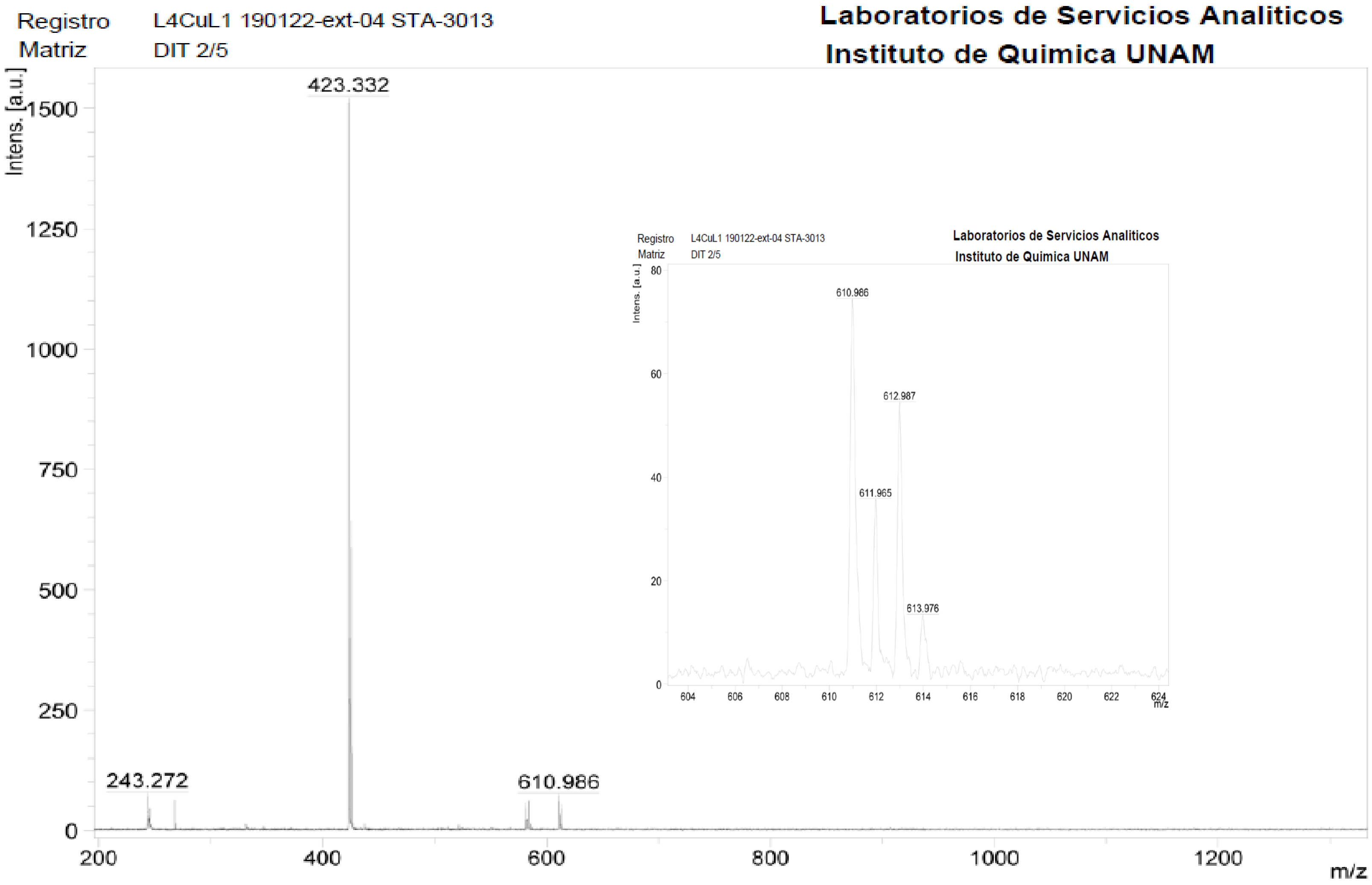

Figure S11. Mass Spectrum D4CuL1.

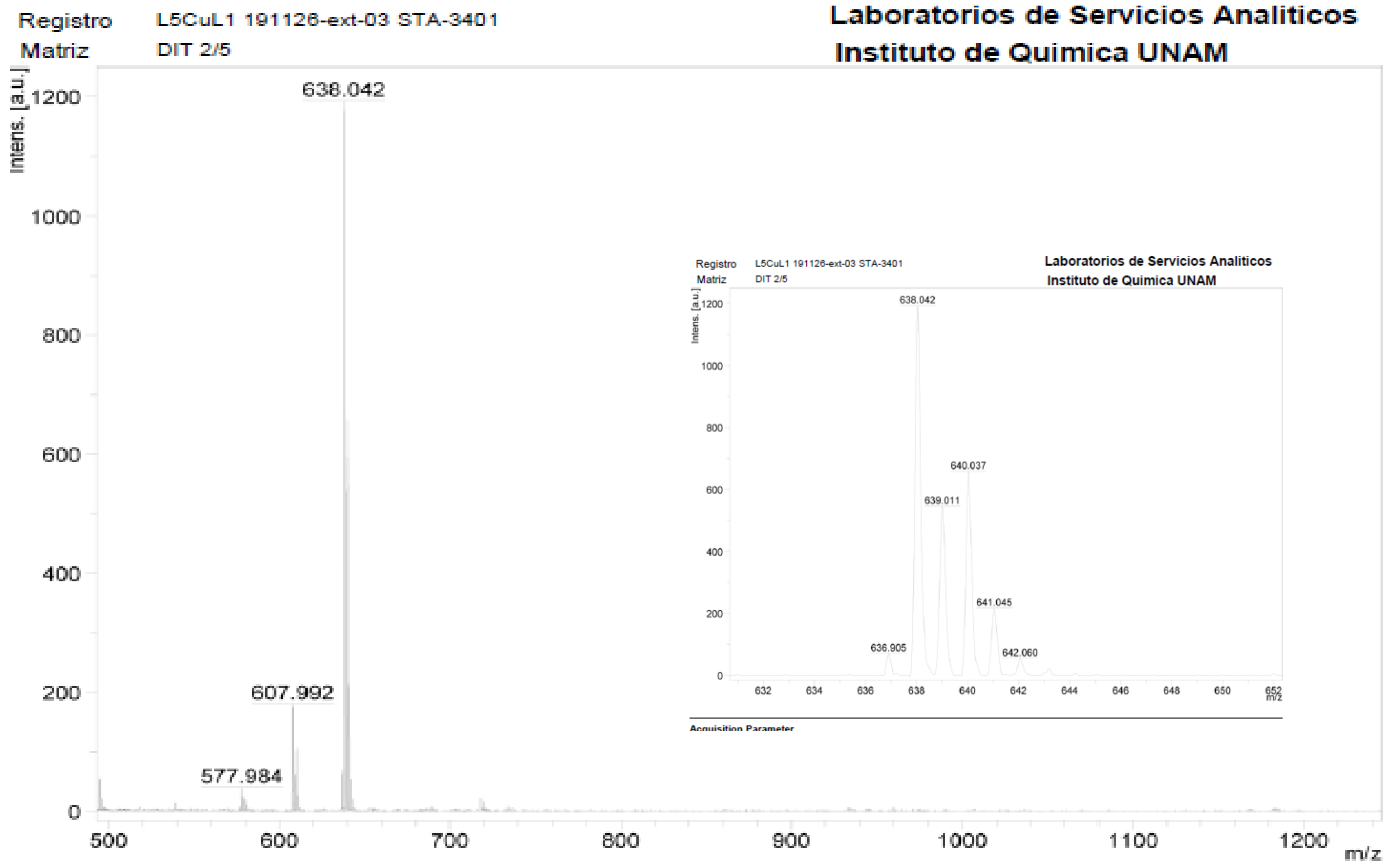

Figure S12. Mass Spectrum D5CuL1.

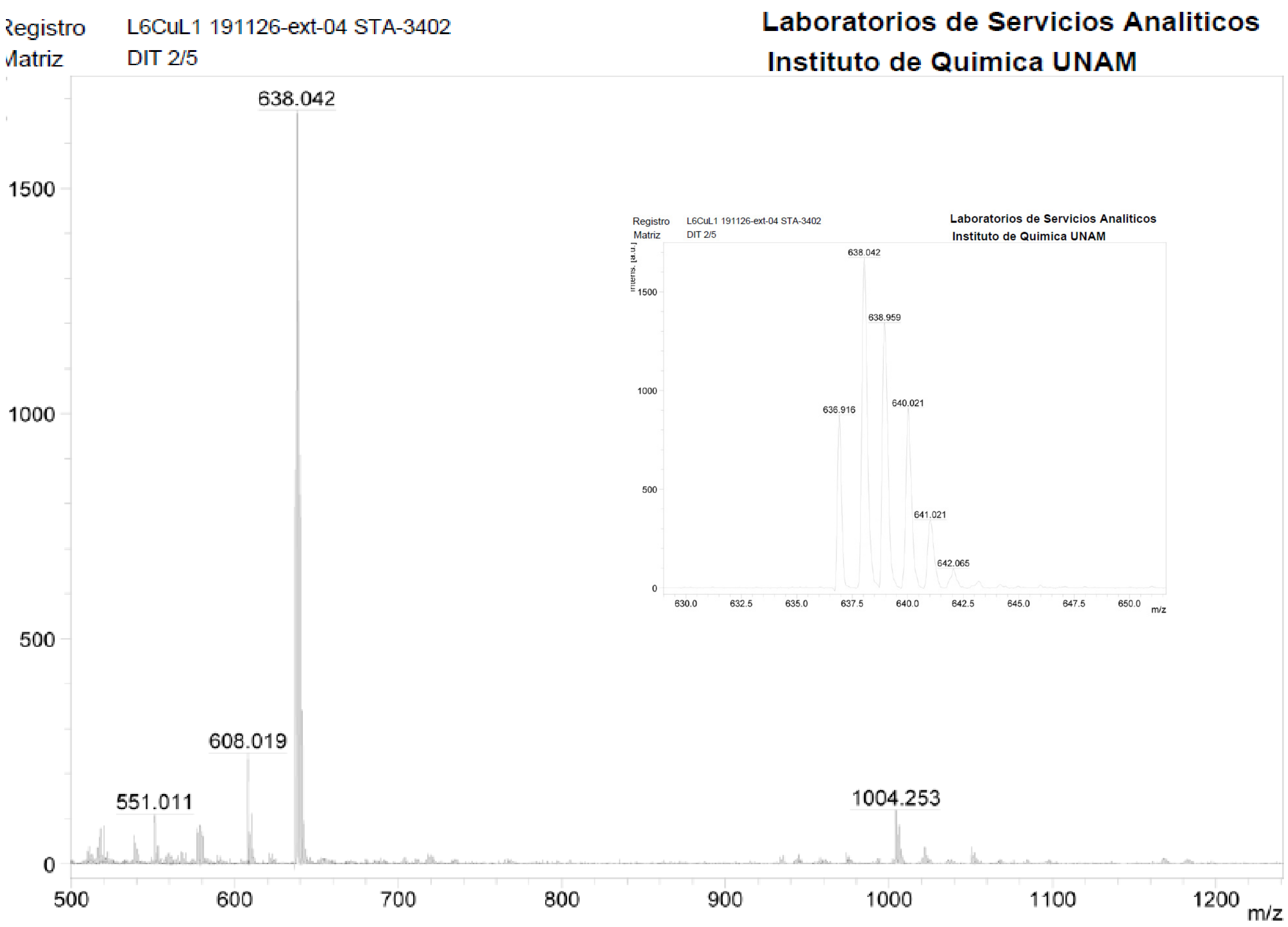

Figure S13. Mass Spectrum D6CuL1.

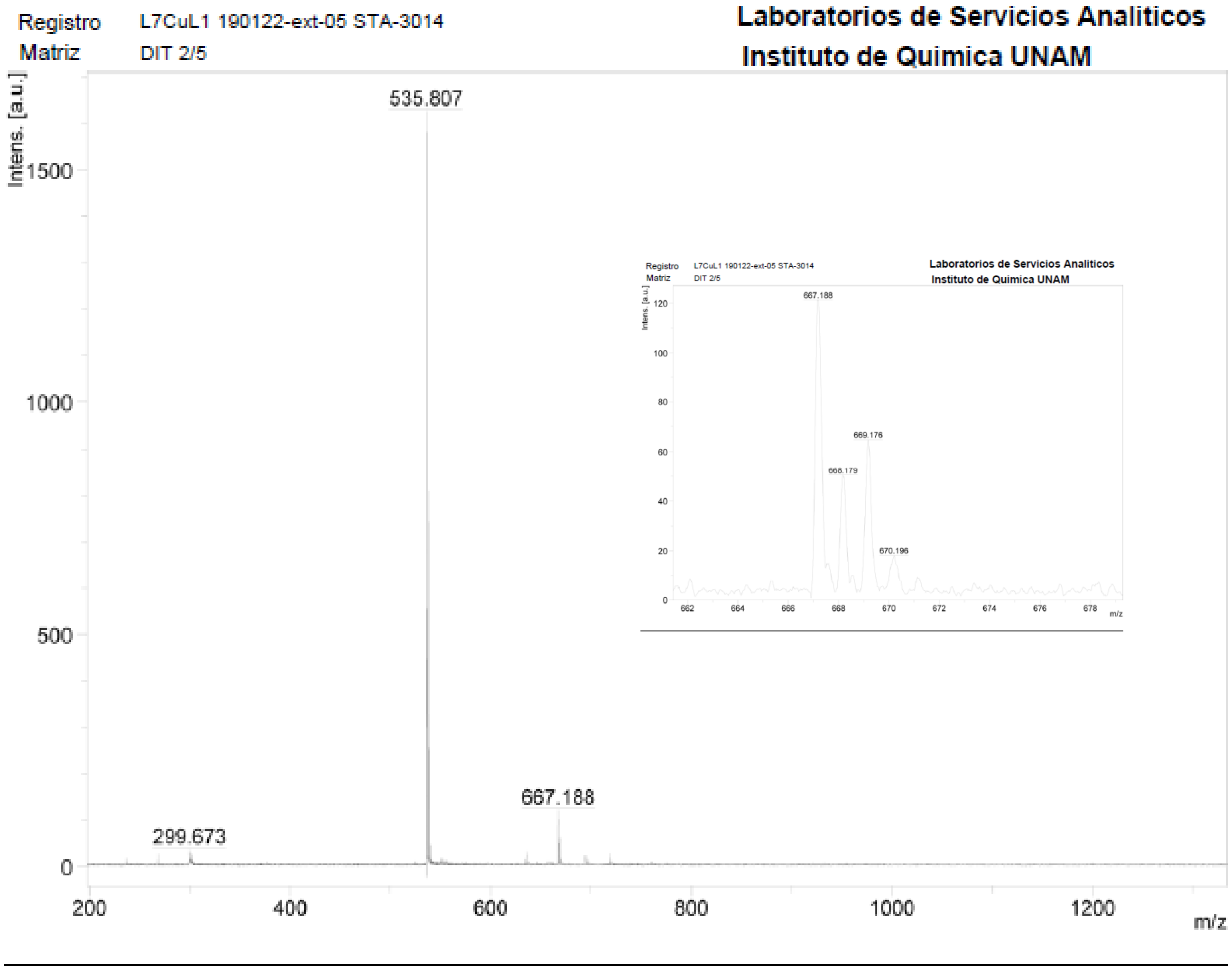

Figure S14. Mass Spectrum D7CuL1.

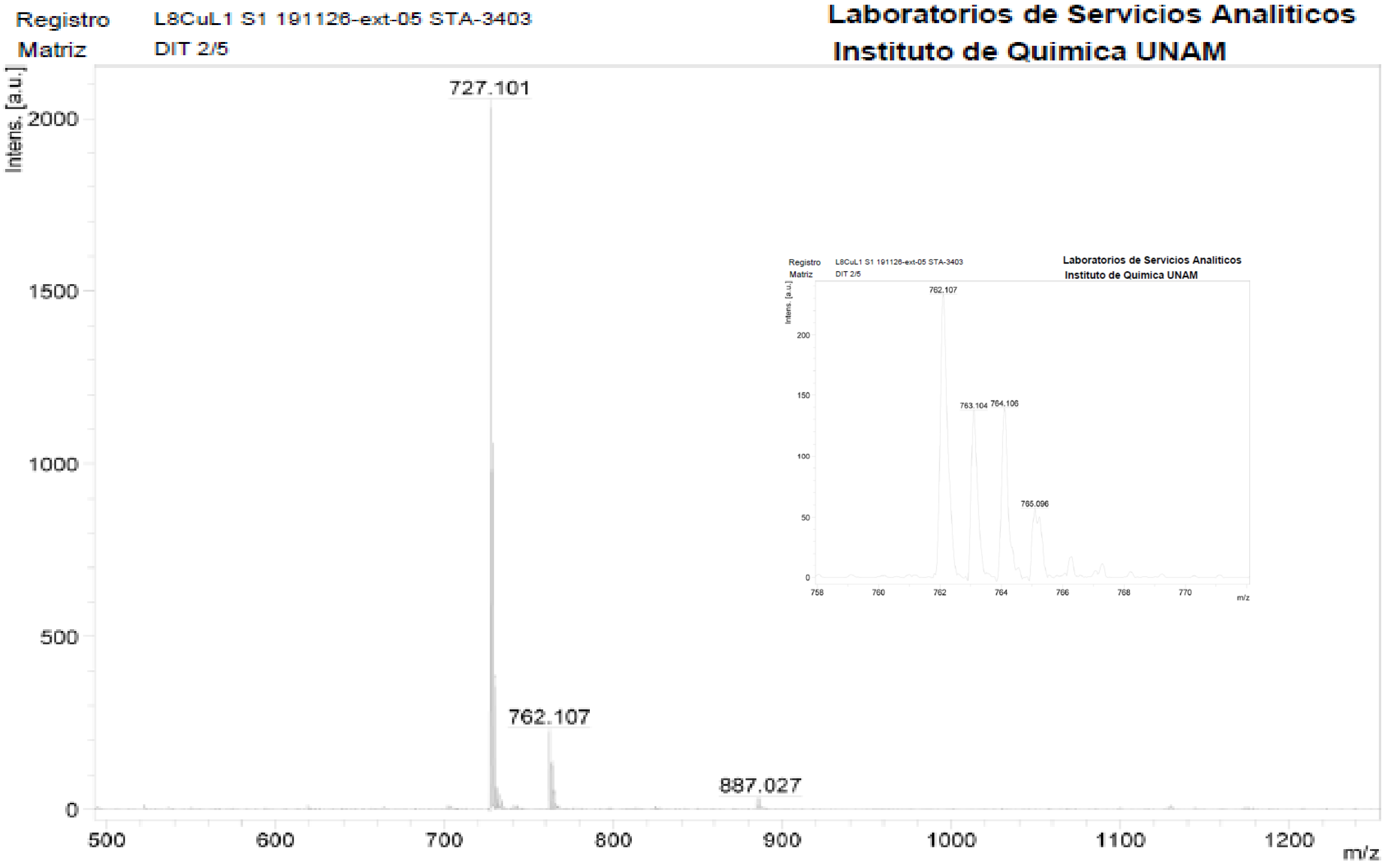

Figure S15. Mass Spectrum D8CuL1.

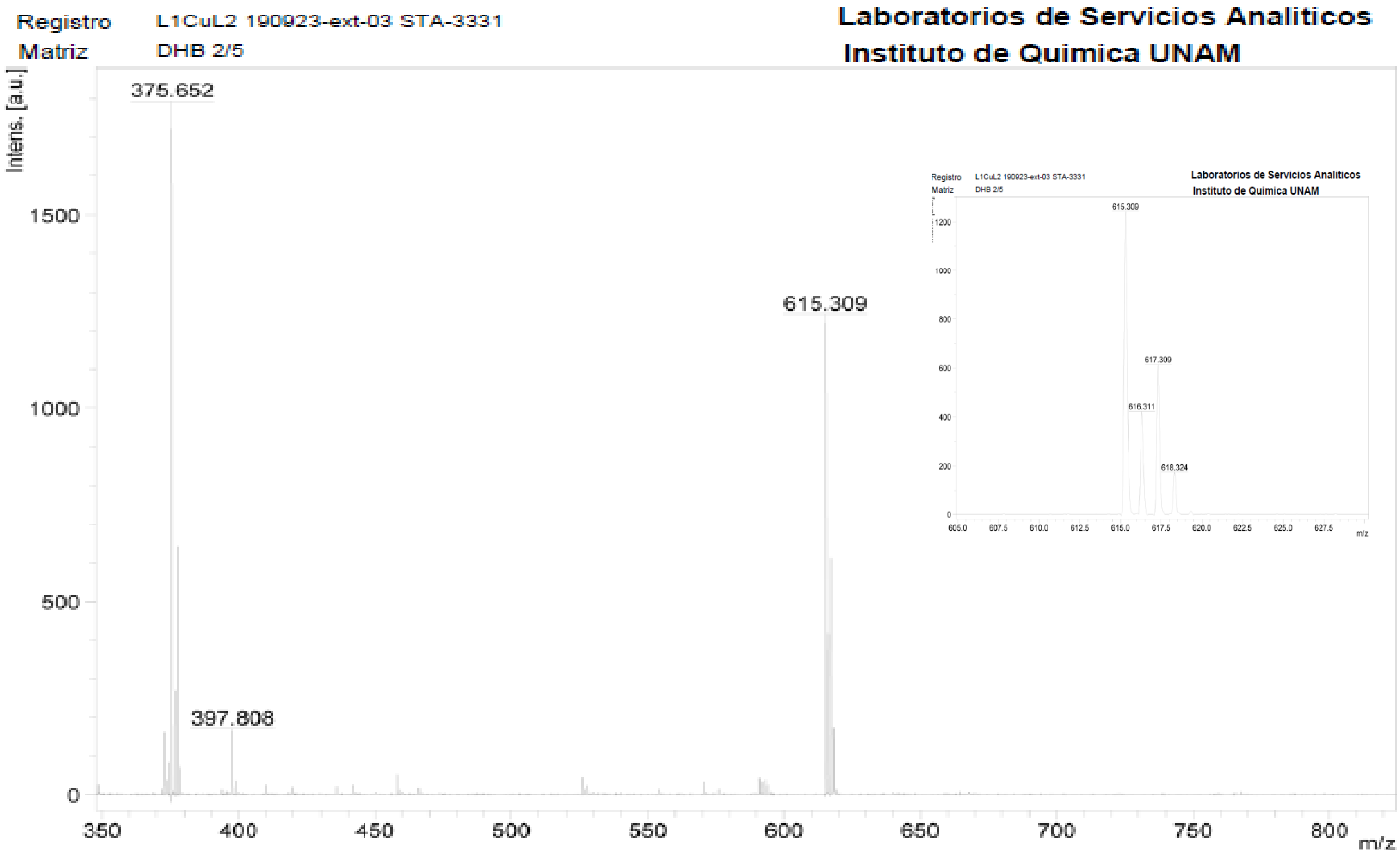

Figure S15. Mass Spectrum D1CuL2

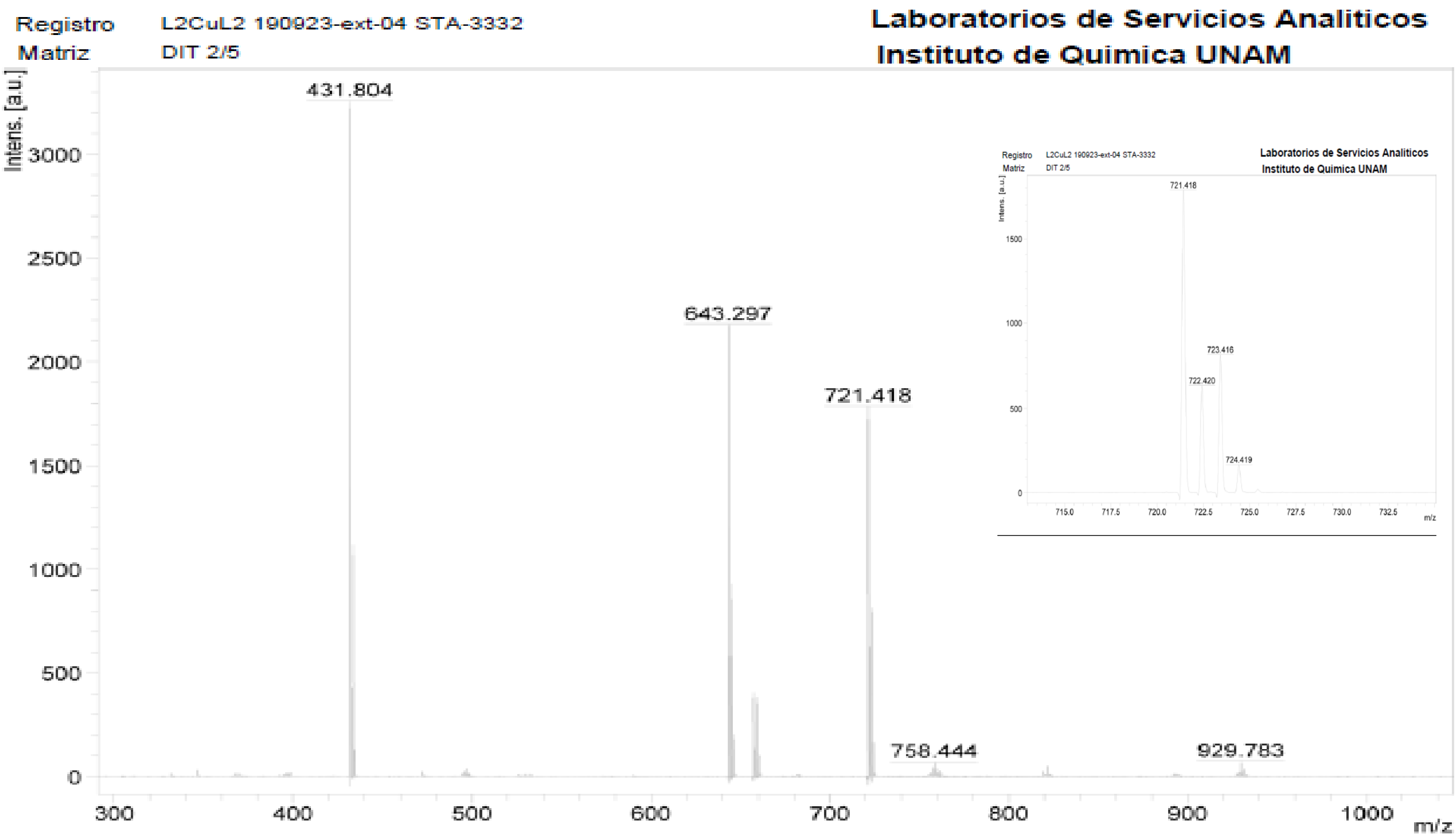

Figure S15. Mass Spectrum D2CuL2

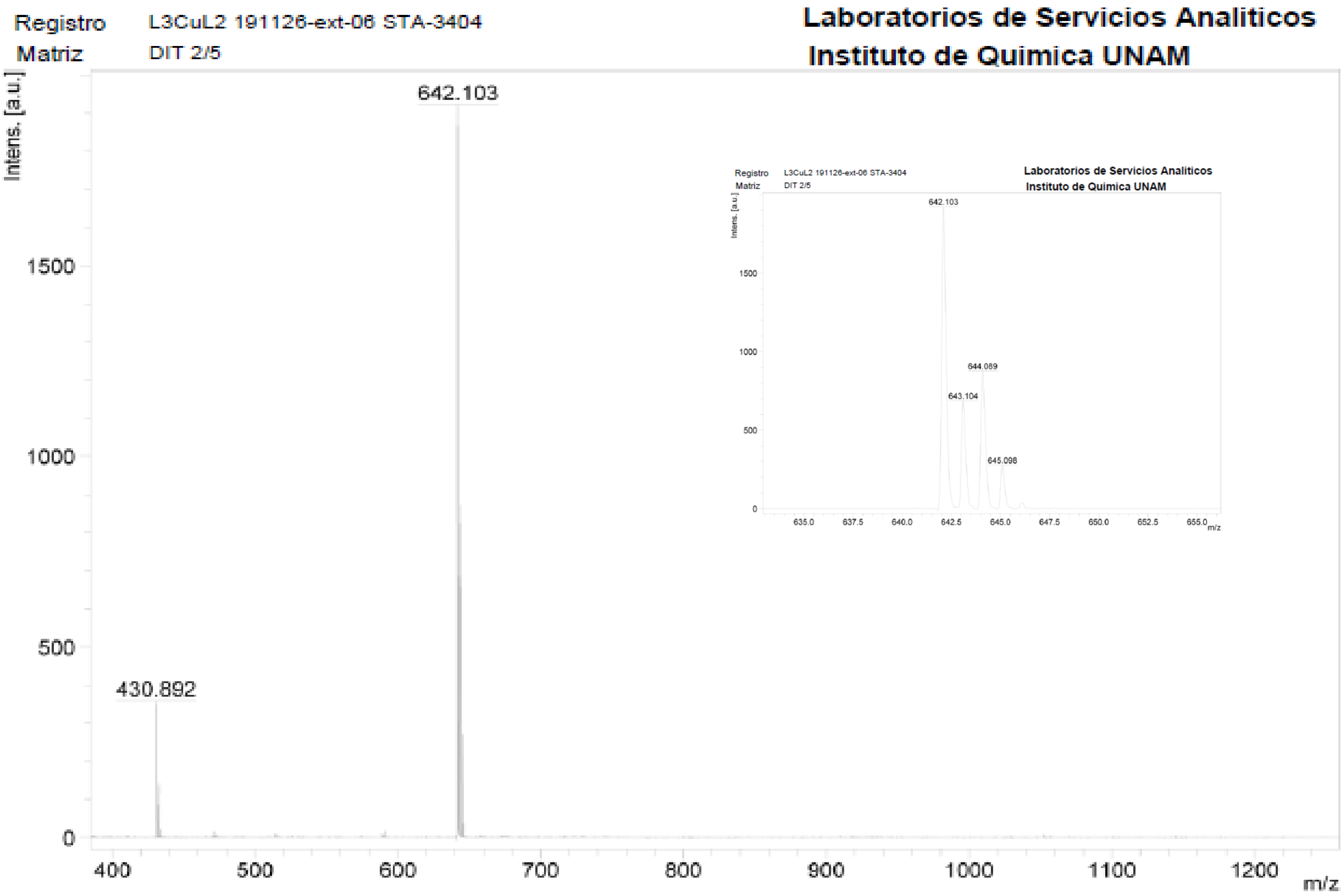

Figure S15. Mass Spectrum D3CuL2

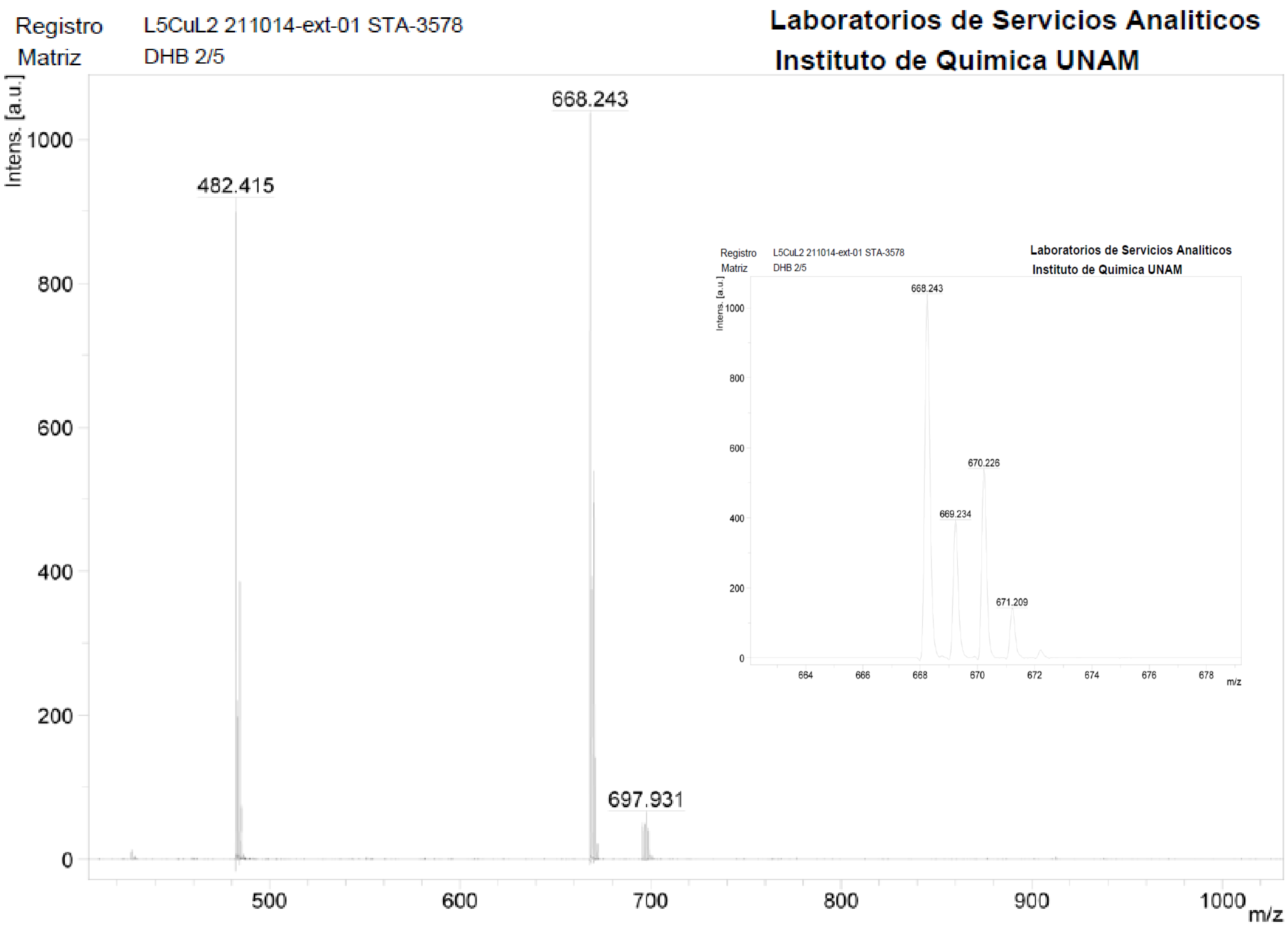

Figure S15. Mass Spectrum D5CuL2

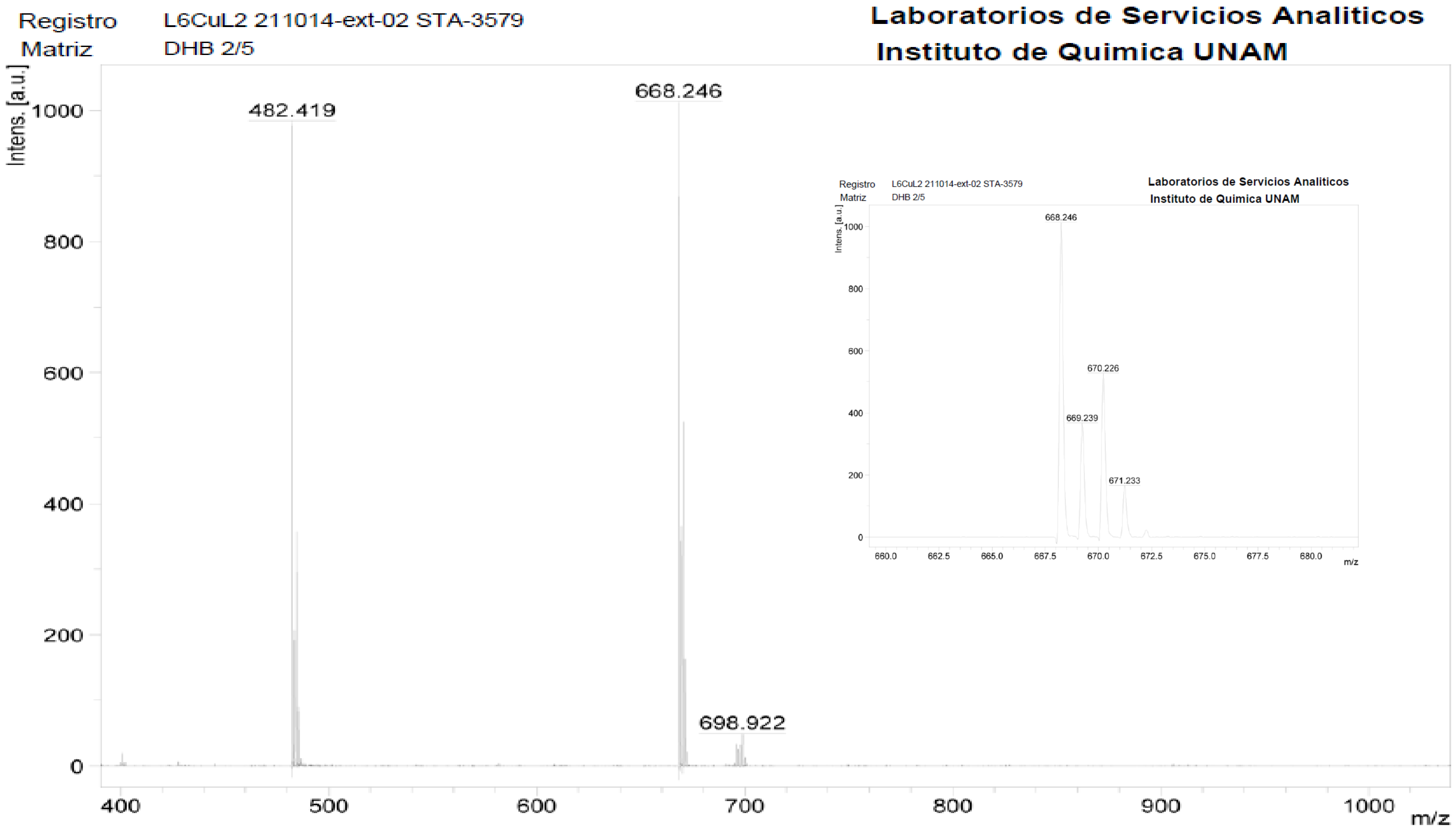

Figure S15. Mass Spectrum D6CuL2

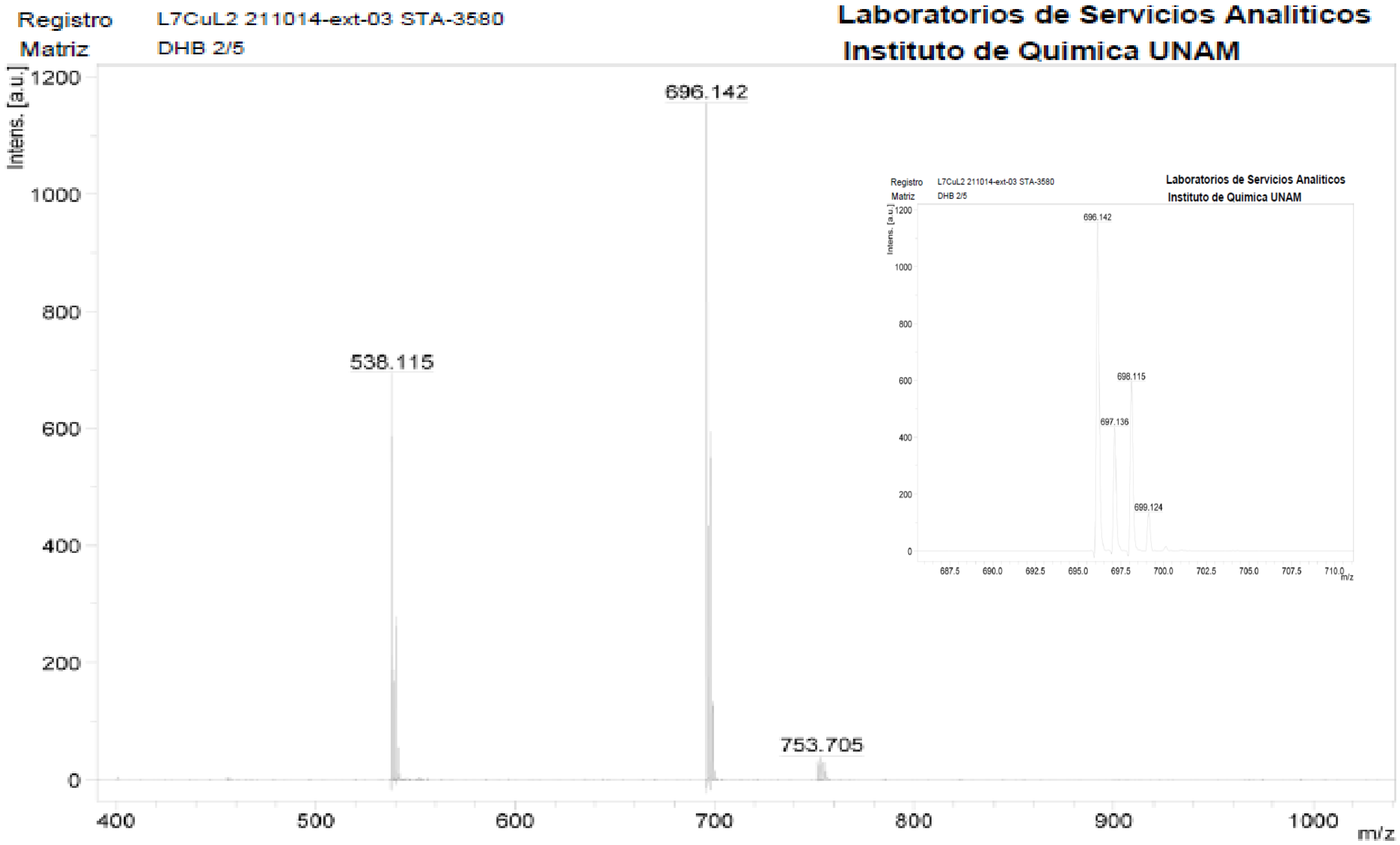

Figure S15. Mass Spectrum D7CuL2

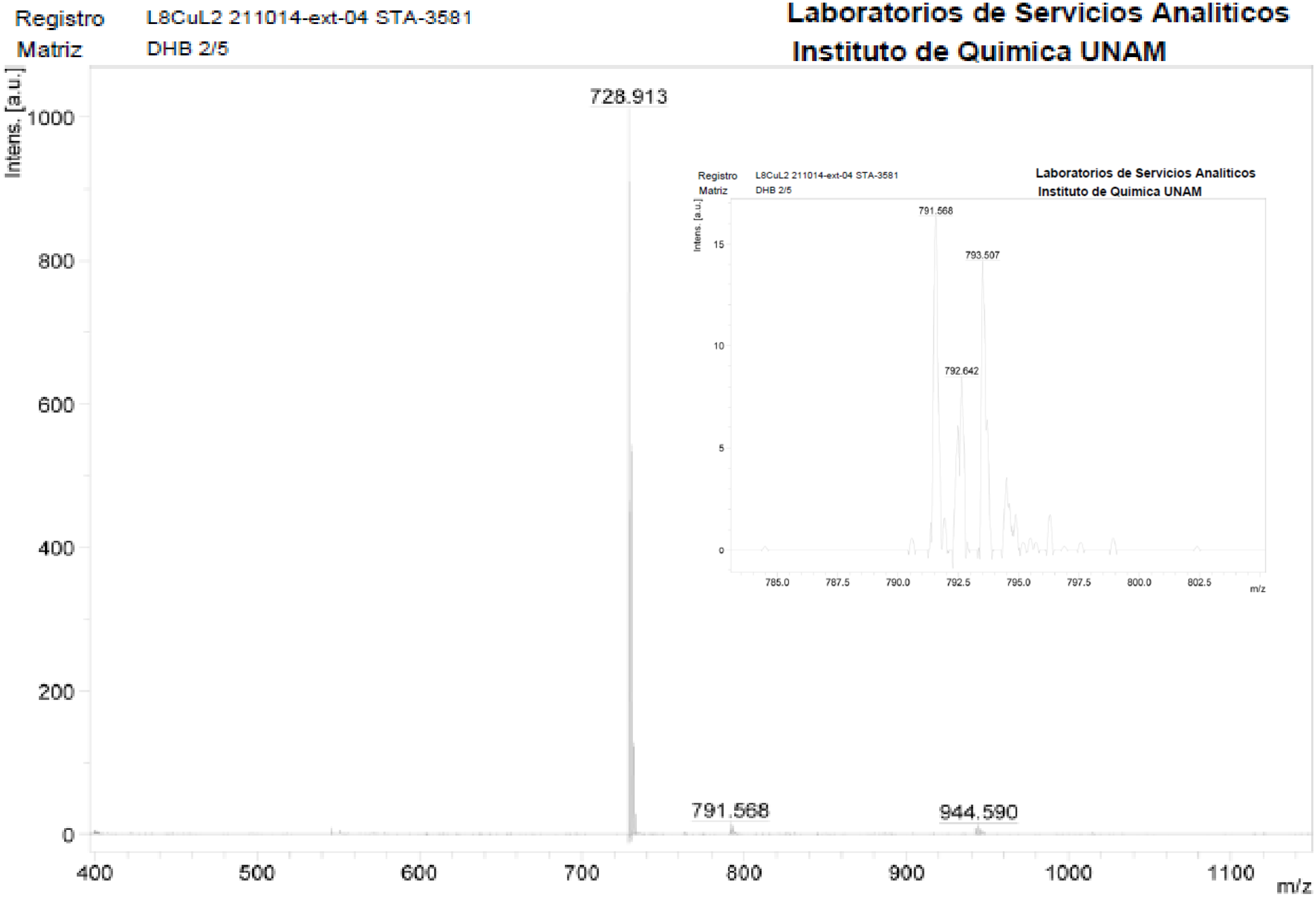

Figure S15. Mass Spectrum D8CuL2

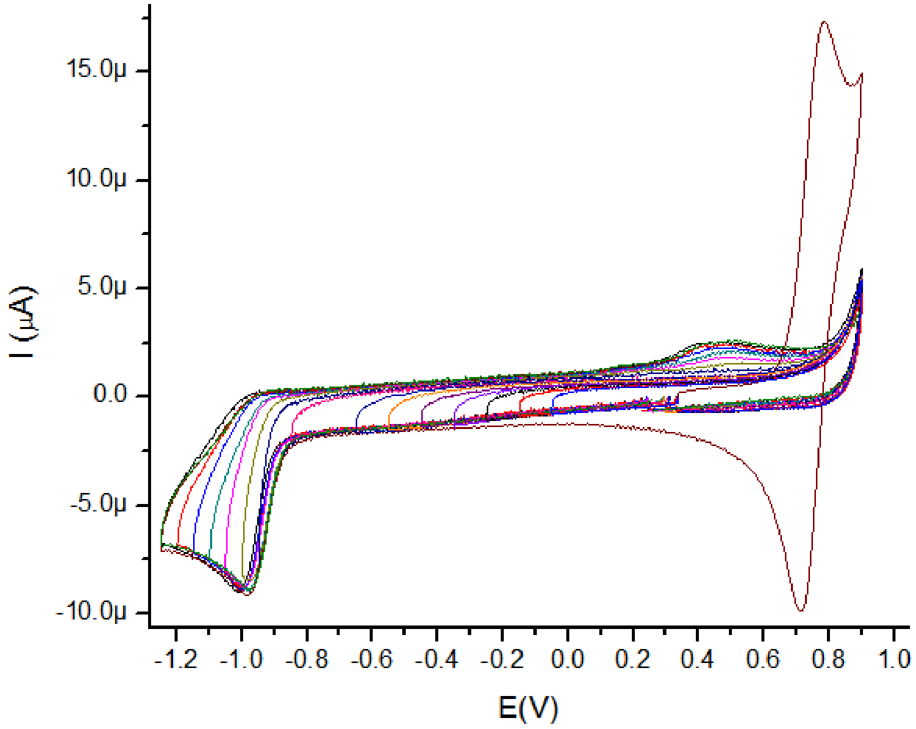

Figure S16. Inversion study of curcumin at 100 mV/s in DMSO. The experiment was referenced to the pair Fc^{+}/Fc.

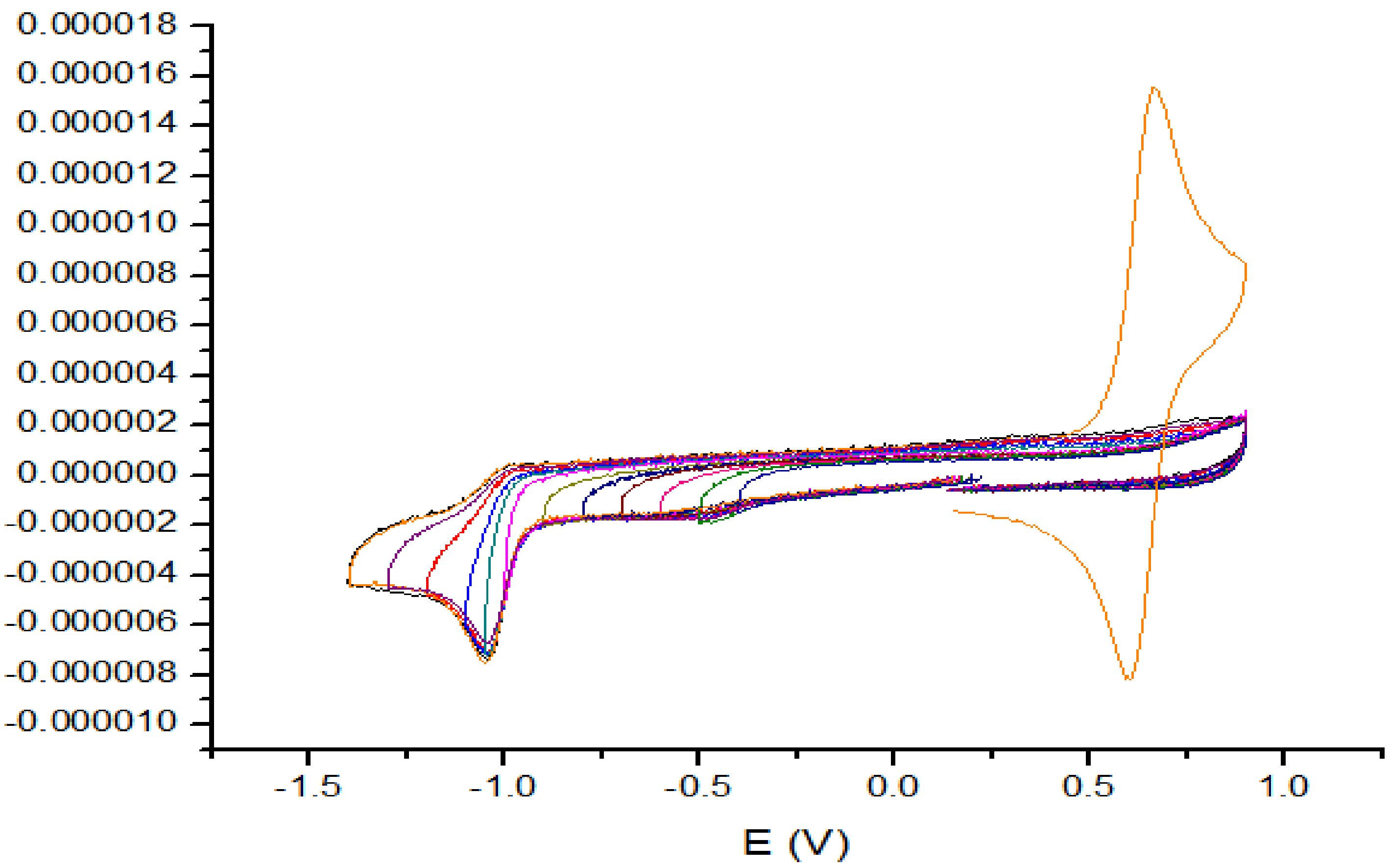

Figure S17. Inversion study of curcumin at 100 mV/s in DMSO. The experiment was referenced to the pair Fc^{+}/Fc.
